# Acute stimulation of PBMCs drives switch from dopamine-induced anti- to proinflammatory phenotype of monocytes only in women

**DOI:** 10.1186/s13293-025-00689-5

**Published:** 2025-02-03

**Authors:** Leonie Fleige, Silvia Capellino

**Affiliations:** Department of Immunology, Research Group of Neuroimmunology, IfADo-Leibniz Research Centre for Working Environment and Human Factors, Ardeystraße 67, 44139 Dortmund, Germany

**Keywords:** Dopamine, Sex-specific differences, Healthy human subjects, Monocytes, B cells, Cytokines, Neuroimmunology

## Abstract

Several studies report an impact of the neurotransmitter dopamine (DA) on human immune cells, with effects dependent on the immune cell type addressed and their activation status. Another contributing factor appears to be sex, as sex-specific differences in the dopaminergic pathway are described in the neurological context as well as in autoimmune diseases. However, a deeper understanding of these differences in peripheral immune cells remains limited. In this study, we investigated the effects of dopaminergic stimulation on activation and cytokine secretion of peripheral blood mononuclear cells from women and men using flow cytometry, ELISA, and multiplex assays. We found a B cell-driven downregulation in cytokine secretion of monocytes exclusively from women under physiological conditions in vitro. Moreover, B cells from men showed higher dopamine receptor (DR) expression, which was shown to be further increased by sex hormones only in men. In monocytes from women, an acute inflammatory stimulus via CpG combined with dopaminergic stimulation caused a switch to a proinflammatory phenotype, which was less pronounced in men. These novel findings in sex-specific responses to dopaminergic stimulation are crucial for understanding DA’s function in the healthy and activated immune system and provide evidence to treat DA-related pathologies in a sex-specific manner.

**Supplementary Information:**

The online version contains supplementary material available at 10.1186/s13293-025-00689-5.

## Highlights

- Dopaminergic stimulation dampens B cell-dependent cytokine secretion of monocytes from women but not from men under physiological conditions.
- A possible underlying explanation could be the higher expression level of DRD_1_ and DRD_3_ on B cells from men compared to women. The expression of DRs is further upregulated by estrogen and testosterone only in men.
- Acute inflammatory stimulus via CpG ODN2006 drives monocytes into a proinflammatory phenotype, that is strengthened by additive dopaminergic stimulation in a B cell independent manner for women and men.
- Thus, CpG ODN2006 induces a switch from anti- to proinflammatory effects after dopaminergic stimulation only in women.

**Supplementary Information:**

The online version contains supplementary material available at 10.1186/s13293-025-00689-5.

## Plain Language summary

This study looks at how the neurotransmitter dopamine affects the immune system in men and women. Dopamine is known for its role in the brain, but it also influences immune cells. In this study, we focused on uncovering differences in how men’s and women’s immune systems respond to dopamine. We examined immune cells obtained from the blood of both women and men and found that dopaminergic stimulation had different effects depending on sex. In women, dopaminergic stimulation reduced the release of important signaling proteins, called cytokines, from specific immune cells, the monocytes, which help the immune system respond to threats. Thus, women but not men showed an anti-inflammatory response after dopaminergic stimulation. This effect was caused by another type of immune cell called B cells. In men, B cells had more receptors that allow dopamine to bind, and these receptors increased further when sex hormones were present. Additionally, when immune cells from women and men were stimulated not only with dopaminergic agents but also in combination with an inflammatory trigger, the cells became more proinflammatory. These findings show that men’s and women’s immune systems react differently to dopamine. This could help developing better treatments for diseases that are influenced by dopamine, with approaches tailored specifically for men or women.

**Supplementary Information:**

The online version contains supplementary material available at 10.1186/s13293-025-00689-5.

## Introduction

The neurotransmitter dopamine (DA) is well known for its role within the central nervous system (CNS), where it modulates movement, cognition, and reward [1]. Over the past decades, the focus has expanded to elucidate DA’s effects on the peripheral immune system. The initial evidence for DA as an immune regulator emerged from the discovery of dopamine receptors (DR) and other proteins involved in the dopaminergic pathway in immune cells [2–4], as well as in their ability to produce, store and metabolize DA [5–7]. DA can bind two types of DRs, D_1_-like (DRD_1_ and DRD_5_) and D_2_-like receptors (DRD_2_, DRD_3_, and DRD_4_). The D_1_-like family typically activates downstream signaling pathways in the brain including an increase in cAMP levels, whereas binding of D_2_-like receptors inhibits this signaling [8, 9]. Besides cAMP-involved signaling, there are also other DR pathways in the brain including for example β-arrestin 2 and PI3K-Akt [10, 11]. It is suggested, that the dopaminergic pathways in peripheral immune cells may differ from the ones in the brain and are not fully discovered yet [1, 12].

The effects of DA on peripheral immune cells are diverse and very context-dependent, ranging from suppression to activation. For example, while DA activates resting effector T cells, it inhibits them once activated [13]. Of note, most studies have focused on T cells, describing DA’s effects on differentiation [14], migration [15, 16], cytokine secretion [17], and proliferation [18], leaving the effects on other peripheral blood mononuclear cell (PBMC) subsets like B cells or monocytes less explored. The rare studies about B cells report increased activation marker expression [3] but contrary effects on proliferation due to DR stimulation [3, 19, 20]. As a modulator of monocytes, DA increased their migration and adhesion [21, 22], while it decreased LPS-induced proliferation [23]. While human monocyte-derived macrophages showed a DA-induced increase in IL6 and MCP1 with and without LPS stimulation [24], in murine macrophages and monocytes, a suppression of LPS-induced IL12p40 production and TNF release was observed [25, 26]. Interestingly, interactions between different immune cell subsets appear to influence DA-induced immune responses [27–29]. For example, the interplay of T_FH_ cells and B cells enhances germinal center output [28], while DA released by monocyte-derived dendritic cells can influence T cell differentiation [29].

Detailed investigations into DA-induced changes in activation status including activation marker expression as well as cytokine secretion in a culture of mixed PBMCs remain limited.

One reason for the diverse and partly contradictory findings could be the influence of sex, which is rarely considered in studies examining DA’s function on peripheral immune cells. In the brain, it is already suggested that the neurotransmitter can act in a sex-specific manner [30]. Evidence that sex hormones can influence the dopaminergic pathway is seen in a change of DRD_1_ density in the brain throughout the female cycle in rats [31]. Furthermore, male rats showed higher DRD_1_ density in the striatum than females [32]. Also in the periphery, healthy men exhibited increased DRD_1_ expression on B cells compared to women [3]. Interestingly, Lee et al. have reported a half palindromic estrogen responsive element on the promotor of DRD_1_ [33] giving a potential epigenetic reason for a connection between the DR pathway and sex hormones. Nevertheless, the impact of sex on the dopaminergic pathway has been little studied yet, especially in the periphery.

Beyond sex, inflammation and pathological conditions also significantly impact DA’s mode of action. Dysregulated DA signaling is associated with various diseases, ranging from CNS-related diseases with an involvement of the peripheral immune system, like multiple sclerosis and Parkinsons disease, to autoimmune diseases, like systemic lupus erythematosus and rheumatoid arthritis (RA) or viral infections like with Human Immunodeficiency Virus (HIV). In Parkinson’s disease, a deficit in DA is characteristic [1, 34, 35], which is caused by the degeneration of dopaminergic neurons due to chronic inflammation in the brain [7, 35, 36]. A strong association with dopaminergic dysfunction is also shown for HIV, including fluctuations in DA levels in the brain throughout the disease progression [37] as well as a DA-induced increase of virus entry into macrophages [38]. In RA or appropriate animal models, DR expression on diverse immune cells is correlated with disease activity [3, 39–41] and DR stimulation affects cytokine secretion, leading to Th17/Treg imbalance and reduced ROS production [41, 42]. In a previous study, we investigated the influence of sex and pathological conditions by stimulating the D_1_-like pathway in PBMCs from female and male healthy donors and RA patients and found a sex-specific increase in IL8 and CCL3 secretion after D_1_-like stimulation only for RA women compared to healthy women but not for men [3].

These findings underline the high clinical relevance to understand DA’s effects in more depth with a special focus on sex-specific differences in health and disease.

Our in vitro study addresses the impact of dopaminergic stimulation on PBMCs under physiological as well as inflammatory conditions, with a detailed focus on cytokine secretion and expression of activation markers on monocytes, but also B cells and T cells from women and men. We found a DA-induced anti-inflammatory phenotype of monocytes under physiological conditions only for women, while DR stimulation supported proinflammatory effects when applied together with CpG, in both sexes but stronger pronounced in women. Under physiological conditions, these effects on monocytes were B cell-driven, whereas inflammation increased the activation of monocytes, thus probably enabling the response to DR agonists without B cell help.

## Material and methods

### Healthy subjects and blood donation

26 healthy women and 21 healthy men participated in this study and gave signed consent. Exclusion criteria were acute or previous hepatitis B or HIV infection, cancer diagnosis and diseases affecting the lung, liver, lymphatic-, cardiovascular-, or nervous system. The recruited female subjects completed a questionnaire regarding their use of hormonal contraception. Only women who were not breastfeeding were included. For blood sampling, lithium heparin collection tubes were used, and the collected blood was processed within 1 h after sampling. An overview of healthy blood donor characteristics is provided in Table 1. Used samples of women and men were age matched for each experiment.

**Table 1:** Overview of healthy blood donor characteristics

	Female			Male
Number	26			21
Age range (years)	21–65			21–72
Average age (years ± SD)	39.2 (± 15.2)			40.2 (± 14.9)
Use of hormonal contraception?	Yes (n = 9)	No (n = 14)	Unknown (n = 3)	n.a

A flow chart of all experiments performed is provided in Supplementary Fig. 1A.

### Isolation of PBMCs and stimulation of cells

After blood sampling, peripheral blood mononuclear cells (PBMCs) were separated from other components of the blood via density gradient centrifugation with Pancoll^™^ (PAN, #P04-60500). For storage, cells were frozen in fetal bovine serum (FBS, Gibco, #10270-106) supplemented with 10% dimethyl sulfoxide (Merck, #472301-1L-M). After thawing, cells were washed with medium (RPMI 1640 medium (PAN, #P04-16515) with 10% FBS, 1% Penicillin–Streptomycin (Gibco, #15140-122), 1% sodium pyruvate (Gibco, #11360-070), 1% MEM non-essential aminoacids (Gibco, #11140-035), 0.1% HEPES (Cytiva, #SH30237.01), 1.918 µl β-mercaptoethanol (Carl Roth, #4227.3)). For stimulation, 200,000 cells per condition were seeded in 100 µl of medium into 96-well round bottom plates, and placed at 37 °C, 5% CO_2_ for two hours, before 100 µl of respective stimulants were added.

### Reagents used for stimulation

For D_1_-like stimulation, A68930 hydrochloride (Tocris, #1534) 10^–7^, 10^–8^ or 10^–9^ M was added to the cells as indicated in the respective Figure Legends. For D_2_-like stimulation DR agonist Ropinirole (Tocris, #3680) was used at concentrations of 10^–7^, 10^–8^ or 10^–9^ M. An acute inflammatory stimulation was induced by the simultaneous addition of 0.195 µM CpG ODN2006 (Invivogen, #tlrl-2006) binding TLR9 as previously shown [39]. For stimulation of sex hormone receptors, 10^–8^, 10^–9^ or 10^–10^ M β-Estradiol (E2, Tocris, #2824/100) or 10^–7^, 10^–8^ or 10^–9^ M 5α-Androstan-17β-ol-3-on (DHT, Sigma Aldrich, #10300-1G-F) was used. The concentrations chosen for the stimulants were based on previous publications.

### Depletion of CD19^+^ B cells and CD14^+^ monocytes

To deplete CD19^+^ B cells or CD14^+^ monocytes, PBMCs were incubated with FBS, purified anti-human CD19 antibody (Biolegend, #302202) or purified anti-human CD14 antibody (Biolegend, #399202) and Mouse IgM (BD Pharmingen^™^, #555,583) in depletion buffer (DPBS (Gibco, #14190-094), containing 2 mM EDTA (Carl Roth, #8043.2) and 2% FBS, sterile filtered) for 20 min at 4 °C. Dynabeads^™^ Pan Mouse IgG (Invitrogen, #11041) were magnetically separated for 1 min and washed with depletion buffer. After incubation, PBMCs were washed, beads were added to the cell suspension, and mixed under rotation for 15 min at RT. The tube containing the cell suspension was placed into the Dynal MPC^®^-15 magnet (Dynal Biotech, #120.29) for 3 min. The cells that were not attached to the beads were collected. The beads were resuspended in depletion buffer and separated a second time. The obtained depleted PBMC fractions were placed again into the magnet for separation, followed by counting of the cells with CASY counter. The efficacy of depletion was analyzed by staining for CD19 or CD14 via flow cytometry. Samples were used only if the remaining proportion of the depleted subtype was less than 1%. The gating strategy is displayed in Supplementary Fig. 1B.

### Quantification of secreted cytokines: ELISA and multiplex assay

To quantify secreted IL8 and MCP1 in the supernatant of complete PBMCs and Monocyte- or B cell-depleted PBMCs, ELISA was performed: ELISA MAX^™^ Standard Set Human IL-8 (Biolegend, #431501) and ELISA MAX^™^ Standard Set Human MCP-1/CCL2 (Biolegend, #438807). The assays were performed as described in the guidelines of the kit, except for the downscaling of all volumes by a factor of two. For quantification of IL1β and IL18 in the supernatant of complete PBMCs and monocyte-depleted PBMCs, LEGENDplex^™^ Human Inflammation Panel 1 (Biolegend, #740809) was used. The measurement was performed with undiluted samples and the protocol was followed as described in the kit guidelines but with each volume reduced by a factor of three. The samples were measured using BD LSRFortessa^™^ flow cytometer.

### Quantification of sex hormones

Within 1 h after blood donation, blood of healthy donors was centrifuged, and plasma was frozen at – 80 °C for later analysis of basal levels of sex hormones using Testosterone Parameter Assay Kit (R&D, #KGE010) and 17β-Estradiol high sensitivity ELISA kit (Enzo Life Sciences, #ADI-900-174). Plasma was diluted 1:6 for measurement of estradiol and 1:10 for measurement of testosterone.

### Flow cytometry staining of dopamine receptors, sex hormone receptors and activation markers including Annexin V

After stimulation, supernatant of PBMCs or depleted fractions was stored at – 80 °C for following cytokine analysis. Cultured or freshly thawed cells were washed with PBS. To stain for dead cells, they were incubated with Zombie UV^™^ Fixable Viability Dye (1:1000, Biolegend, #423108) in PBS for 20 min at 4 °C in the dark and washed afterwards. PBS containing 2% Albumin Fraction V (Carl Roth, #0163.4) was added to each condition to block unspecific binding sites for 20 min at 4 °C in the dark. Staining of extracellular markers in staining buffer (PBS supplemented with 2% FBS) followed for 20 min at 4 °C. After washing, cells, that were previously stained with activation marker panel, were incubated with FITC Annexin V (1:1600, Biolegend, #640906) in Annexin V Binding Buffer (Biolegend, #422201) for 15 min at RT in the dark. Cells, that were previously stained with dopamine receptor or sex hormone receptor panel, were incubated with staining buffer containing 2% paraformaldehyde (Aldrich, #16005) for 10 min at RT in the dark and washed again. For permeabilization, FACS^™^ Permeabilizing Solution 2 (diluted 1:10 with dH_2_O, BD, #340,973) was added and incubated for 10 min at RT in the dark. Cells were washed, unspecific binding sites were blocked, and intracellular antigens were stained. After washing, cells were measured with the Cytek^®^ Aurora (5 Lasers) flow cytometer. The list of antibodies can be found in supplementary Table 1.

### Intracellular cytokine measurement via flow cytometry

After thawing and stimulation of PBMCs, Brefeldin A (1:1000, Sigma Aldrich, #B7651-5MG) was added to the cell culture medium directly with applied stimulation reagents or 18 h after initial stimulation. Staining of dead cells, blocking of unspecific binding sites and staining of extracellular markers was conducted as described in 2.7. Afterwards, PBMCs were fixed with 2% paraformaldehyde, permeabilized with FACS^™^ Permeabilizing Solution 2, and unspecific binding sites were blocked with 2% Albumin Fraction V as described in 2.7. Intracellular cytokines were stained for 20 min at RT in the dark. After washing, samples were measured at the Cytek^®^ Aurora (5 Lasers) flow cytometer. The list of antibodies can be found in supplementary Table 2.

### Software and statistical analysis

FlowJo (Version 10.3) was used to analyze flow cytometry data, and Prism 9 (GraphPad Software, v 9.2.0) statistical analysis. For illustration, Prism 9 (GraphPad Software, v 9.2.0) and BioRender.com (2020) were utilized. Each figure legend mentions the statistical analyses that were used for every experiment. Single measurements were performed in flow cytometry experiments, duplicates in ELISA and multiplex measurements. Each graph represents the mean with standard error.

## Results

### Cytokine secretion of monocytes is reduced by DR stimulation only in women

**Fig. 1:**
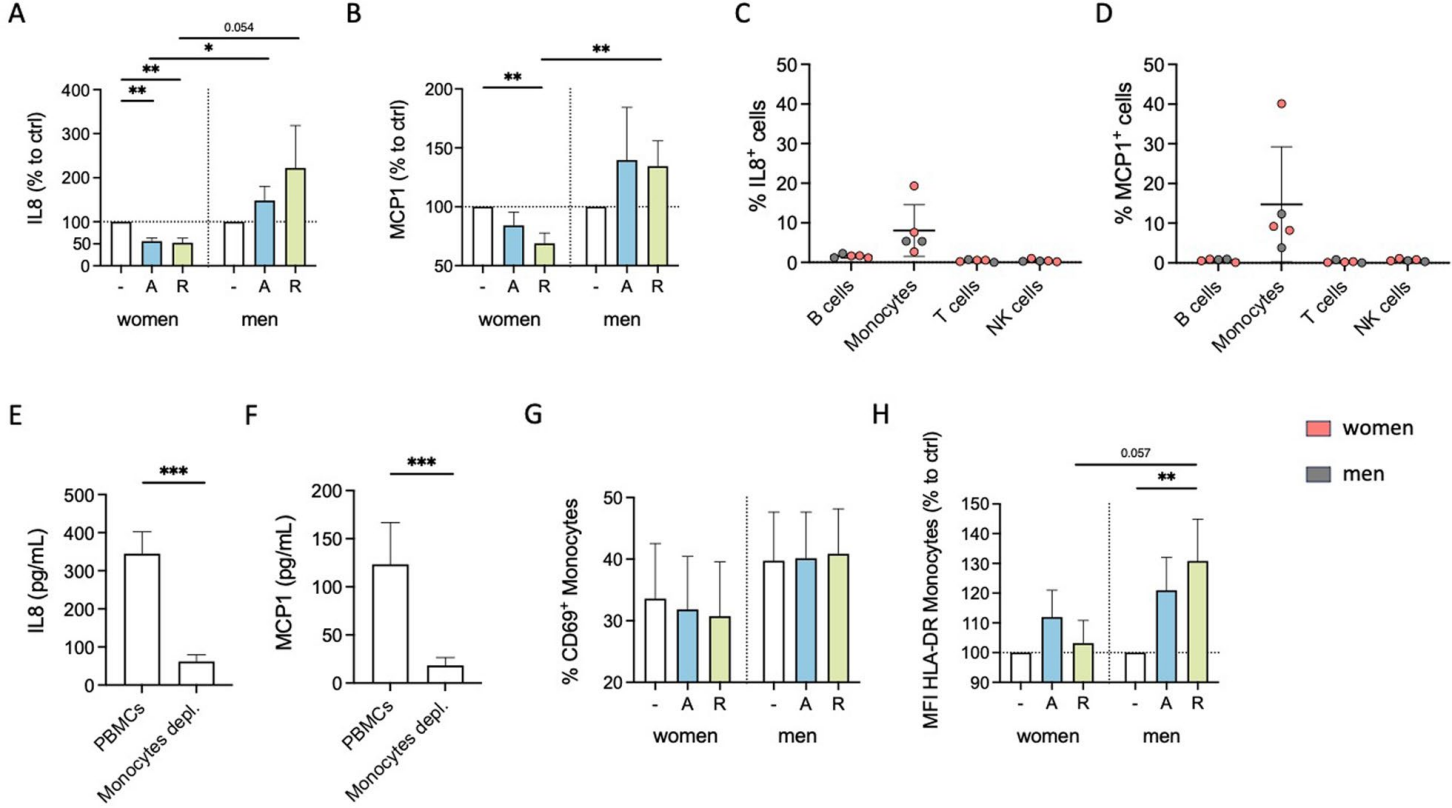
DR stimulation reduces physiological cytokine secretion and activation marker expression of monocytes from women compared to men. **A**, **B** IL8 (**A**) and MCP1 (**B**) levels in supernatant from PBMCs of women and men after 24 h in cell culture, with and without in vitro stimulation by A68930 (A, 10^–7^ M) or Ropinirole (R, 10^–6^ M) measured via ELISA; n = 11–13 per group; normalized to unstimulated control. Basal levels are presented in Supplementary Fig. 2A and B. **C**, **D** Percentage of IL8^+^ (**C**) and MCP1^+^ (**D**) B cells, monocytes, T cells, and NK cells after 24 h in culture without stimulation measured via flow cytometry; n = 5 per subtype. **E**, **F** IL8 (**E**) and MCP1 (**F**) levels in supernatant from mixed PBMCs and CD14^+^ monocyte-depleted PBMCs after 24 h in culture without stimulation measured via ELISA; n = 11–13 per condition. **G**, **H** Percentage of CD69^+^ monocytes (**G**) and expression of HLA-DR on monocytes (**H**) from women and men after 24 h in culture of mixed PBMCs, with and without stimulation by A68930 (A, 10^–7^ M) or Ropinirole (R, 10^–6^ M) measured via flow cytometry; n = 13–14 per group; normalized to unstimulated control. Basal levels are presented in Supplementary Fig. 2G and H. Mann–Whitney test was used for comparing unpaired data of women and men, and Wilcoxon test for comparing paired data including unstimulated vs. stimulated samples as well as mixed PBMCs vs. monocytes depleted PBMCs; *p ≤ 0.05, **p ≤ 0.01, ***p ≤ 0.001

Cytokine secretion is an important immune-modulating property since it leads to the regulation of inflammatory responses and the coordination of cellular communication within the immune system [43, 44]. We investigated whether stimulation with the D_1_-like agonist A68930 or the D_2_-like agonist Ropinirole can influence the cytokine secretion of PBMCs from healthy women and men under physiological conditions in vitro. IL8 and MCP1 were detected at measurable levels under physiological conditions (Supplementary Fig. 2A and B), whereas other cytokines were below the detection limit (measured via LEGENDplex^™^ Human Inflammation Panel 1; data not shown). Upon D_1_- and D_2_-like stimulation, there was a significant decrease in IL8 and MCP1 secretion by PBMCs from women. At the same time, this was not observed in men (Fig. 1A and B, Supplementary Fig. 2C and D).

Previously, we already showed that DRD_1_, DRD_2_, DRD_3_ and DRD_4_, but not DRD_5_, are expressed on all PBMC subsets, namely B cells, T cells, NK cells, and monocytes [3], thus, being potentially responsible for the observed changes in cytokine secretion. Intracellular IL8 and MCP1 staining (Fig. 1C and D) and depletion of monocytes from PBMCs showing a strong reduction of these secreted cytokines (Fig. 1E and F) confirmed that their source are the monocytes. Furthermore, DR stimulation itself did not induce IL8 or MCP1 production in B cells, T cells or NK cells (Supplementary Fig. 2E and F).

Additionally, we investigated whether DR stimulation affected the activation status of monocytes, as indicated by the percentage of CD69^+^ monocytes and the expression of activation markers HLA-DR, CD86, and CD38. While we did not observe a significant impact of dopaminergic stimulation on CD69-expressing monocytes (Fig. 1G) or the expression of CD86 and CD38 (Supplementary Fig. 2I-L) in both sexes, D_2_-like stimulation resulted in an upregulation of HLA-DR on monocytes from men but not on monocytes from women (Fig. 1H).

### B cells from male subjects have a higher expression level of DRD_1_ and DRD_3_ than B cells from women

**Fig. 2:**
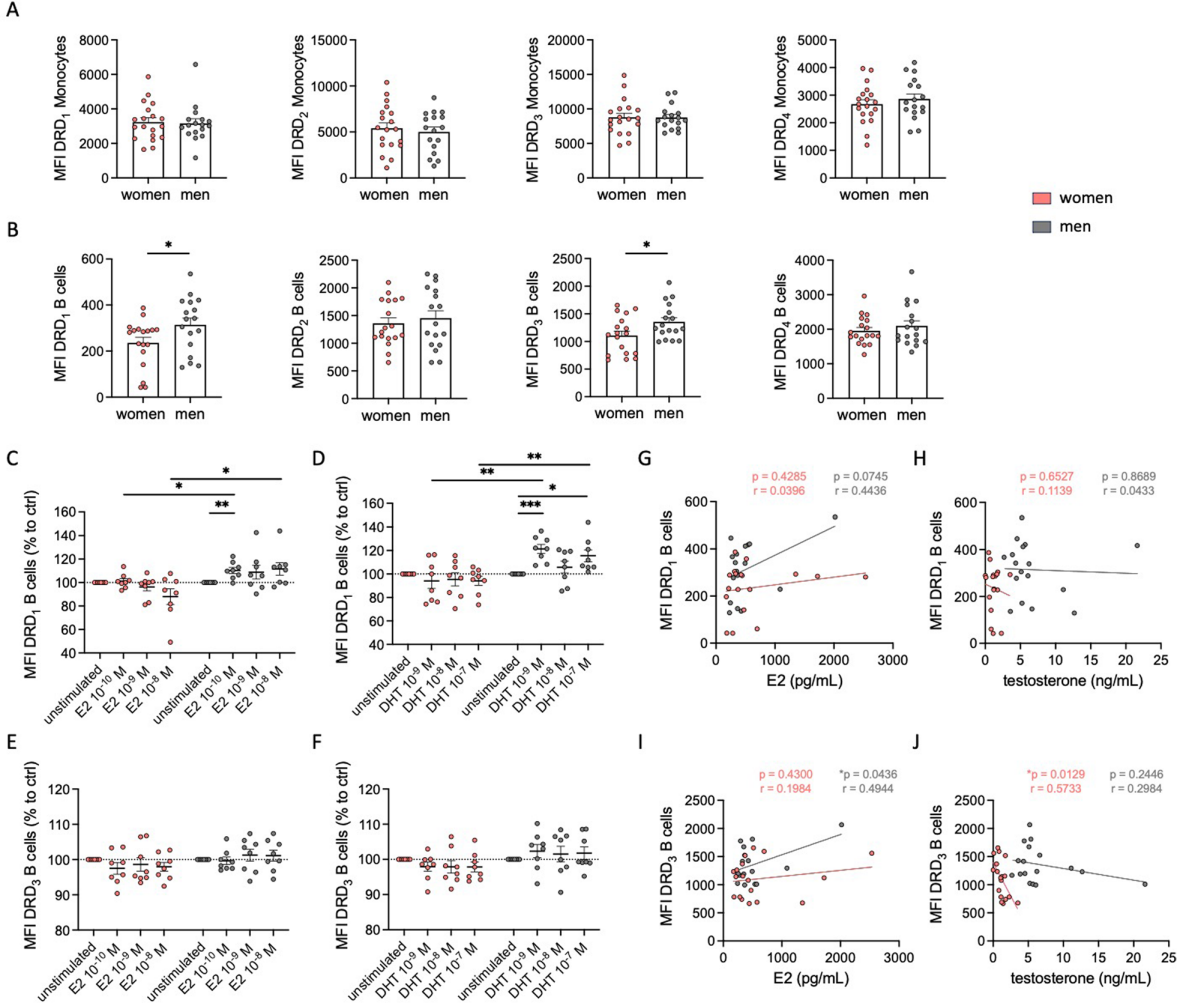
Higher DRD_1_ and DRD_3_ expression in B cells from men was partly regulated by estradiol and testosterone. **A**, **B** Basal expression of DRD_1_, DRD_2_, DRD_3,_ and DRD_4_ on monocytes (**A**) and B cells (**B**) from women and men measured via flow cytometry; n = 17–19 per group. **C**, **D** DRD_1_ expression on B cells from women and men after 24 h stimulation with E2 (10^–8^, 10^–9^, 10^–10^ M, C) and DHT (10^–7^, 10^–8^, 10^–9^ M, D) in mixed PBMC culture measured via flow cytometry and normalized to DMSO control; n = 8 per condition. **E**, **F** DRD_3_ expression on B cells from women and men after 24 h stimulation with E2 (10^–8^, 10^–9^, 10^–10^ M, E) and DHT (10^–7^, 10^–8^, 10^–9^ M, F) in mixed PBMC culture measured via flow cytometry and normalized to DMSO control; n = 8 per condition. **G**, **H** Correlation of DRD_1_ expression on B cells from women and men with basal estrogen (**G**) and testosterone (**H**) levels in plasma; n = 17–18 per group.** I**, **J** Correlation of DRD_3_ expression on B cells from women and men with basal estrogen (**I**) and testosterone (**J**) levels in plasma; n = 17–18 per group. Women: orange; men: grey. Unpaired t-test was used for comparing women and men, while simple linear regression was used to analyze the correlation of DR expression with sex hormone levels. One-way ANOVA with Geisser-Greenhouse correction and Dunnett multiple comparisons test was used for statistical testing of sex hormone receptor (SHR) stimulation with three concentrations of E2 and DHT. Mann–Whitney test compared the effects of SHR stimulation between women and men at the same hormone concentrations; *p ≤ 0.05, **p ≤ 0.01, ***p ≤ 0.001

Based on these sex-specific effects of DR agonists on cytokine secretion, we hypothesized that the differences could be attributed to a sex-specific expression of DRs on PBMCs. To test this hypothesis, we analyzed the expression patterns of DRs via flow cytometry. All DRs except DRD_5_ were found on all PBMC subsets (data not shown). These results are in line with previous findings, except of DRD_5_, which was described on immune cells in other studies [2, 22]. Consistent with a previous study [3], we did not observe significant differences in the expression of DRs on monocytes between women and men (Fig. 2A). However, higher expression levels of DRD_1_ and DRD_3_, but not DRD_2_ and DRD_4_, were found in B cells from men compared to women (Fig. 2B).

Other studies have already reported that the expression of DRs seems to correlate with the level of sex hormones [31–33]. Thus, we stimulated PBMCs with 17β-estradiol (E2) and dihydrotestosterone (DHT) and looked for a change in DRD_1_ and DRD_3_ expression on B cells afterwards. We found an upregulation of DRD_1_ on male compared to female B cells (Fig. 2C and D), however, not for DRD_3_ (Fig. 2E and F). This upregulation observed only in men is not related to a higher amount of sex hormone receptors on male B cells, since our flow cytometry analysis did not reveal differences in the expression levels of estrogen receptors ERα, ERβ, and GPR30, as well as androgen receptor AR between both sexes (Supplementary Fig. 3A-D).

Next, we measured E2 and testosterone in the plasma to test for a possible correlation with DR expression. Surprisingly, we found comparable levels of E2 in plasma from both women and men in our cohort (Supplementary Fig. 3E). This finding can be partly explained by the lower E2 levels in women taking hormonal contraceptives (26% of the total cohort) compared to those without (Supplementary Fig. 3F). As expected, women in our cohort exhibited significantly lower testosterone levels compared to men (Supplementary Fig. 3G). The direct effect, that was observed after sex hormone stimulation on DRD_1_ expression, could not be confirmed in correlation analyses between E2 or testosterone levels with DR expression on B cells (Fig. 2G–J), underscoring that the level of sex hormones in plasma cannot be used as a predictor for DR expression on immune cells.

### DR stimulation led to a slight but women-specific increase in B cell activation

**Fig. 3:**
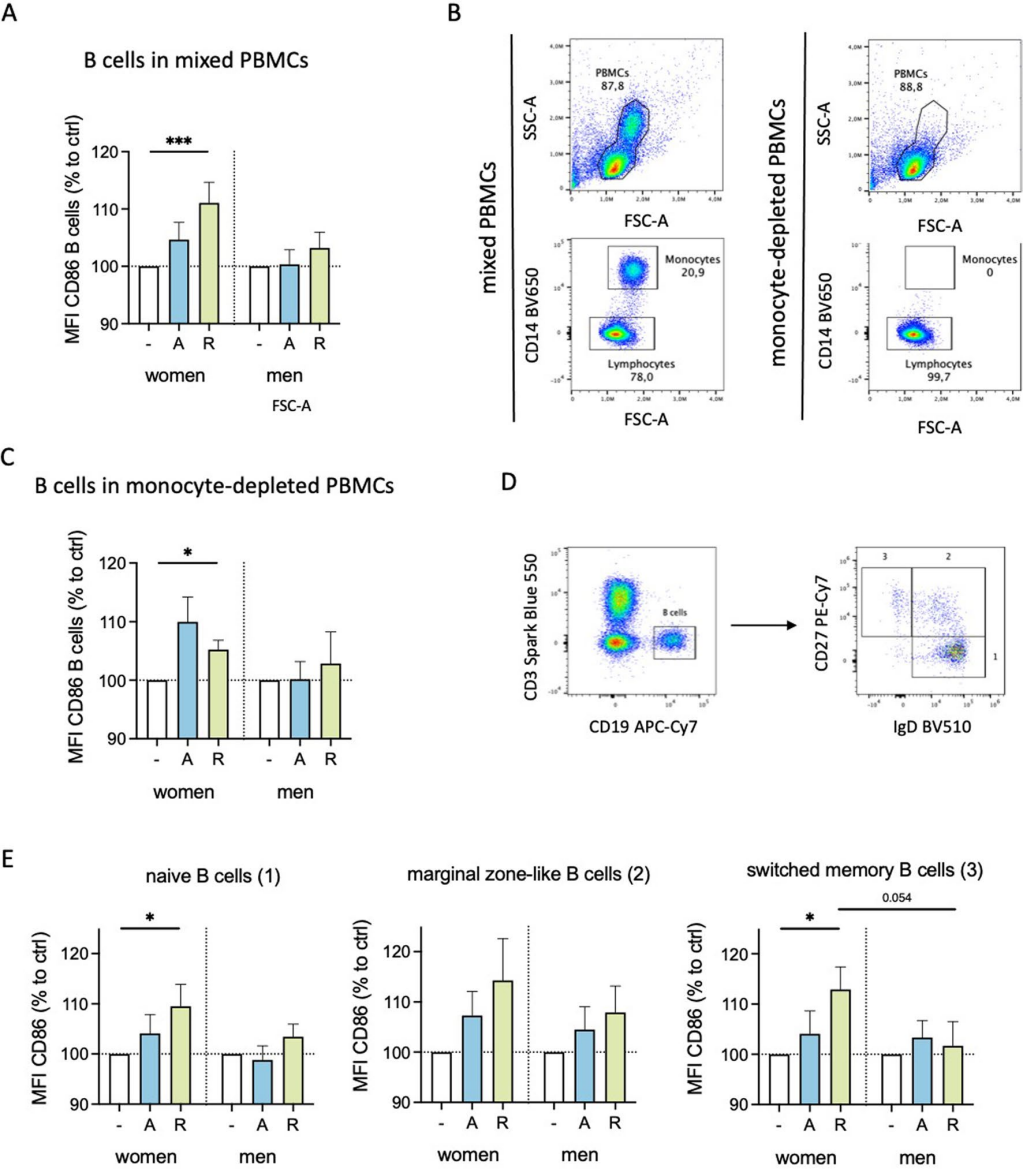
DR stimulation increased activation of female B cells. **A** CD86 expression on B cells from women and men after 24 h in mixed PBMC culture, with and without stimulation by A68930 (A, 10^–7^ M) or Ropinirole (R, 10^–6^ M) measured via flow cytometry; stimulated samples are normalized to unstimulated control; n = 13–14 per group. Basal levels are presented in Supplementary Fig. 4A. **B** Representative flow cytometry plots of complete and CD14^+^ monocyte-depleted PBMCs. **C** CD86 expression on B cells from women and men after 24 h in monocyte-depleted PBMC culture, with and without stimulation by A68930 (A, 10^–7^ M) or Ropinirole (R, 10^–6^ M) measured via flow cytometry; stimulated samples are normalized to unstimulated control; n = 7–8 per group. **D** Gating strategy for B cell subsets (1, naïve B cells: IgD^+^CD27-; 2, marginal zone-like B cells: IgD^+^CD27^+^; switched memory B cells: IgD^−^CD27^+^). **E** CD86 expression on naïve, marginal zone-like, and switched memory B cells from women and men after stimulation of mixed PBMCs with A68930 (A, 10^–7^ M) or Ropinirole (R, 10^–6^ M) for 24 h normalized to unstimulated control measured via flow cytometry; n = 13–14 per group. Mann–Whitney test was used for testing statistical significance between unpaired data of women and men. Wilcoxon test was used for the comparison of paired data including unstimulated vs. stimulated samples; *p ≤ 0.05, ***p ≤ 0.001

Since we observed differently expressed DRD_1_ and DRD_3_ in B cells from women and men, we next investigated whether dopaminergic stimulation influences activation markers on B cells. Interestingly, D_2_-like stimulation significantly increased CD86 on B cells in women but not men, and the same tendency was observed for D_1_-like stimulation (p = 0.094; Fig. 3A), suggesting a higher activated phenotype for B cells from women upon dopaminergic stimulation. While CD69 did not show a significant change after DR stimulation (Supplementary Fig. 4D), HLA-DR was slightly upregulated in both sexes, indicating a not-sex-specific effect (Supplementary Fig. 4E).

To test whether the increased activation of B cells was due to indirect effects mediated by monocytes, we depleted monocytes from the cell culture (Fig. 3B) and examined the effects of dopaminergic stimulation. D_2_-like stimulation still caused an upregulation in CD86 expression exclusively in women (Fig. 3C) as observed for B cells in mixed PBMCs, thus excluding a possible secondary effect mediated through monocytes.

Considering that DR expression in B cells has been reported to correlate with different stages of B cell development [3], we analyzed whether the observed differences in CD86 regulation could be attributed to specific B cell subsets. Thus, we stained for IgD^+^CD27^−^ naïve B cells, IgD^+^CD27^+^ marginal zone-like B cells and IgD^−^CD27^+^ switched-memory B cells (Fig. 3D). All three B cell subsets showed an increase in CD86 expression exclusively in women following dopaminergic stimulation (Fig. 3E).

These findings provide strong evidence for the sex-specific effects of dopaminergic stimulation on B cells.

### B cells contribute to the sex-specific cytokine decrease of female monocytes after DR stimulation

**Fig. 4:**
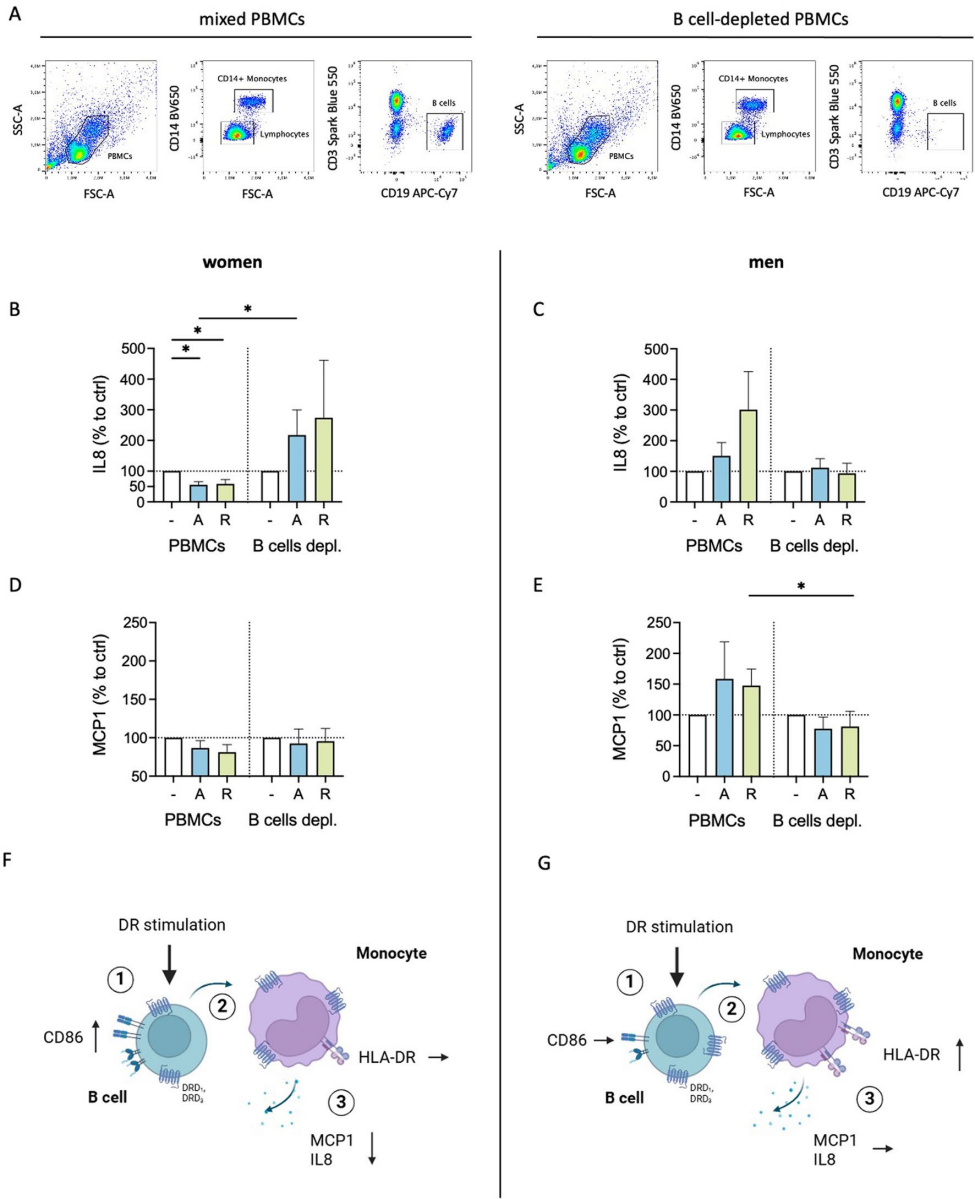
Sex-specific cytokine secretion of monocytes after DR stimulation is dependent of B cells. **A** Representative flow cytometry plots of mixed PBMCs and CD19^+^ B cell-depleted PBMCs.** B**, **C** IL8 levels in supernatant from PBMCs and B cell-depleted PBMCs from women (**B**) and men (**C**) after 24 h in culture, with and without stimulation by A68930 (A, 10^–7^ M) or Ropinirole (R, 10^–6^ M) measured via ELISA; normalized to unstimulated control; n = 8–9 per group. **D**, **E** MCP1 levels in supernatant from PBMCs and B cell-depleted PBMCs from women (**D**) and men (**E**) after 24 h in culture, with and without stimulation by A68930 (A, 10^–7^ M) or Ropinirole (R, 10^–6^ M) measured via ELISA; normalized to unstimulated control; n = 8 per group. **F**, **G** Diagrams illustrating the interplay between B cells and monocytes after DR stimulation, showing effects on activation markers and cytokine secretion for cells from women (**F**) and men (**G**); created in BioRender.com. Wilcoxon test was used for paired data comparisons including PBMCs vs. B cells depleted as well as unstimulated vs. stimulated samples. Mann–Whitney test was used for testing statistical significance between unpaired data of women and men; *p ≤ 0.05

Next, we investigated whether the presence of B cells influences the downregulated cytokine secretion by monocytes observed exclusively for women. To explore this, we depleted B cells from the culture of mixed PBMCs (Fig. 4A) and measured the levels of IL8 and MCP1 in supernatant from B cell-depleted fraction from women and men. The downregulation of IL8 and MCP1 secretion from female monocytes observed in mixed PBMC cultures after stimulation with dopaminergic agents (Fig. 1A and B) was not seen after B cell depletion (Fig. 4B and D). A change in IL8 and MCP1 production also by male monocytes was seen in the absence of B cells (Fig. 4C and E), suggesting a possible B cell-dependent mechanism.

In summary, DR stimulation leads to increased activation of female B cells, which can subsequently induce a reduction in the activation and cytokine secretion of monocytes. Conversely, this activation in B cells in response to DR stimulation is not observed for men, and consequently, there is no downregulation in activation and cytokine secretion of monocytes (Fig. 4F and G).

### Acute stimulation induces switch to proinflammatory effect of DR agonists on cytokine secretion in women

In a previous study, we demonstrated sex-specific responses to DR stimulation in patients with rheumatoid arthritis (RA), a chronic inflammatory disease [3]. Additionally, changes in dopaminergic effects due to the coactivation of the MAPK signaling pathway are reported [45]. To elucidate whether sex-specific differences can also be observed under acute inflammatory conditions, we introduced CpG, which also leads to activated MAPK signaling [46]. We demonstrated that the stimulation with CpG activated both B cells and monocytes without indirect B cell influence (Supplementary Fig. 5A and B).

Our findings showed that, in the presence of CpG, IL8 was still mainly produced by monocytes as shown by the intracellular staining of IL8 (Supplementary Fig. 6A) and the reduction in IL8 level after depletion of monocytes from PBMCs (Supplementary Fig. 6B). However, a CpG-induced increase in IL8 expression has also been found for B cells (Supplementary Fig. 6A). With B cells and monocytes as mixed sources for IL8, we observed a comparable increase in IL8 by CpG stimulation in both sexes, with no effect by dopaminergic stimulation, unlike under physiological conditions, which presented monocytes as the exclusive IL8 source (Supplementary Fig. 6C and D).

**Fig. 5:**
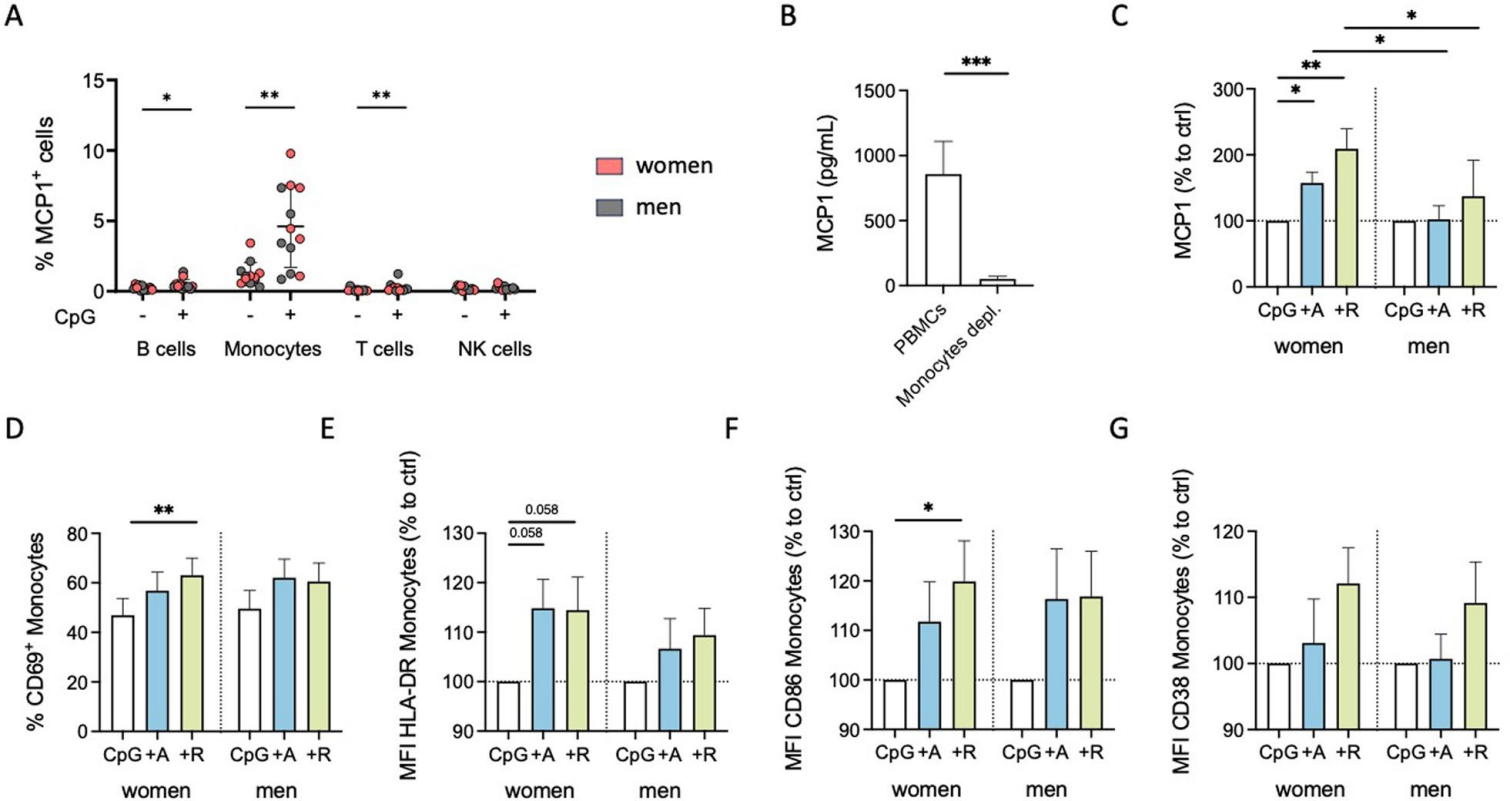
Inflammatory condition turned DR stimulation-induced characteristics of female monocytes into proinflammatory phenotype.** A** Percentage of MCP1^+^ B cells, monocytes, T cells, and NK cells after 24 h in mixed PBMC culture, with or without CpG (0.195 μM) stimulation, measured via flow cytometry; n = 12 per condition.** B** MCP1 levels in supernatant from mixed and monocyte-depleted PBMCs after 24 h of CpG stimulation measured via ELISA; n = 14 per condition.** C** MCP1 levels in supernatant from PBMCs of women and men after 24 h in culture with CpG (0.195 μM) with or without A68930 (A, 10^–7^ M) or Ropinirole (R, 10^–6^ M) measured via ELISA; normalized to CpG control; n = 11–13 per group. Basal levels of unstimulated and CpG stimulated samples are presented in Supplementary Fig. 5C. **D-G** Percentage of CD69^+^ monocytes (**D**) and expression of HLA-DR (**E**), CD86 (**F**) and CD38 (**G**) on monocytes from women and men after 24 h in culture of PBMCs with or without CpG (0.195 μM) stimulation and after stimulation with CpG + A68930 (A, 10^–7^ M) and CpG + Ropinirole (R, 10^–6^ M) measured via flow cytometry; normalized to CpG control; n = 14 per group. Basal levels of unstimulated and CpG stimulated samples are presented in Supplementary Fig. 5D-G. Wilcoxon test was used for paired data comparisons including samples of two different stimulations as well as PBMCs vs. monocytes depleted. Mann–Whitney test was used for testing statistical significance between unpaired data of women and men; *p ≤ 0.05, **p ≤ 0.01, ***p ≤ 0.001

We focused on the secretion of MCP1 since only monocytes and no other PBMC subset showed a detectable level of MCP1-expressing cells, which were upregulated by CpG stimulation after 24 h (Fig. 5A, Supplementary Fig. 6E). Furthermore, the depletion of monocytes resulted in nearly undetectable levels of MCP1 (Fig. 5B), underscoring that monocytes are the sole source of MCP1. Interestingly, CpG stimulation increased MCP1 expression in both sexes; however, combined with dopaminergic agents, an even more pronounced increase was observed only for women but not men (Fig. 5C). Thus, dopaminergic stimulation under inflammatory conditions switched the anti-inflammatory phenotype to a proinflammatory effect regarding MCP1 secretion only in women (Supplementary Fig. 6F and G).

In addition to MCP1, IL1β and IL18 also appeared to be monocyte-specific since the depletion of monocytes led to an almost complete loss of these cytokines in the supernatant (Supplementary Fig. 6H and I). The release of both cytokines was induced by CpG (Supplementary Fig. 6J and K), confirming its activating effects also on monocytes, and was further increased by DR stimulation in women (Supplementary Fig. 6L and M). This supportsthe proinflammatory phenotype of female monocytes due to DR stimulants under these inflammatory conditions. This finding was further strengthened by an increase in CD69^+^ monocytes and the increased expression of activation markers HLA-DR and CD86 on monocytes induced by DR stimulation, which was not statistically significant in men (Fig. 5D–G). Notably, the switch in the effects from physiological to inflammatory conditions was pronounced for the number of CD69-expressing monocytes, but similar for both women and men (Supplementary Fig. 7A and B). This was also evident for CD86 and CD38, resulting in a stronger upregulation after DR stimulation in the presence of CpG than without additional stimulus (Supplementary Fig. 7E–H), but not for HLA-DR (Supplementary Fig. 7C and D).

In summary, our results indicate that the effects of dopaminergic stimulation are shifted to a proinflammatory response regarding cytokine secretion and monocyte activation by an acute inflammatory stimulus specifically in women.

Interestingly, DR stimulation also led to an increase in HLA-DR and CD86 on T cells under physiological as well as inflammatory conditions (Supplementary Fig. 8) suggesting an increased activation for this PBMC subtype. However, these effects on T cells were independent of sex and the presence of an acute inflammatory stimulus, presenting the observed responses of B cells and monocytes to be cell specific.

### Monocytes exhibit a B cell-independent proinflammatory phenotype after DR stimulation under inflammatory conditions

**Fig. 6:**
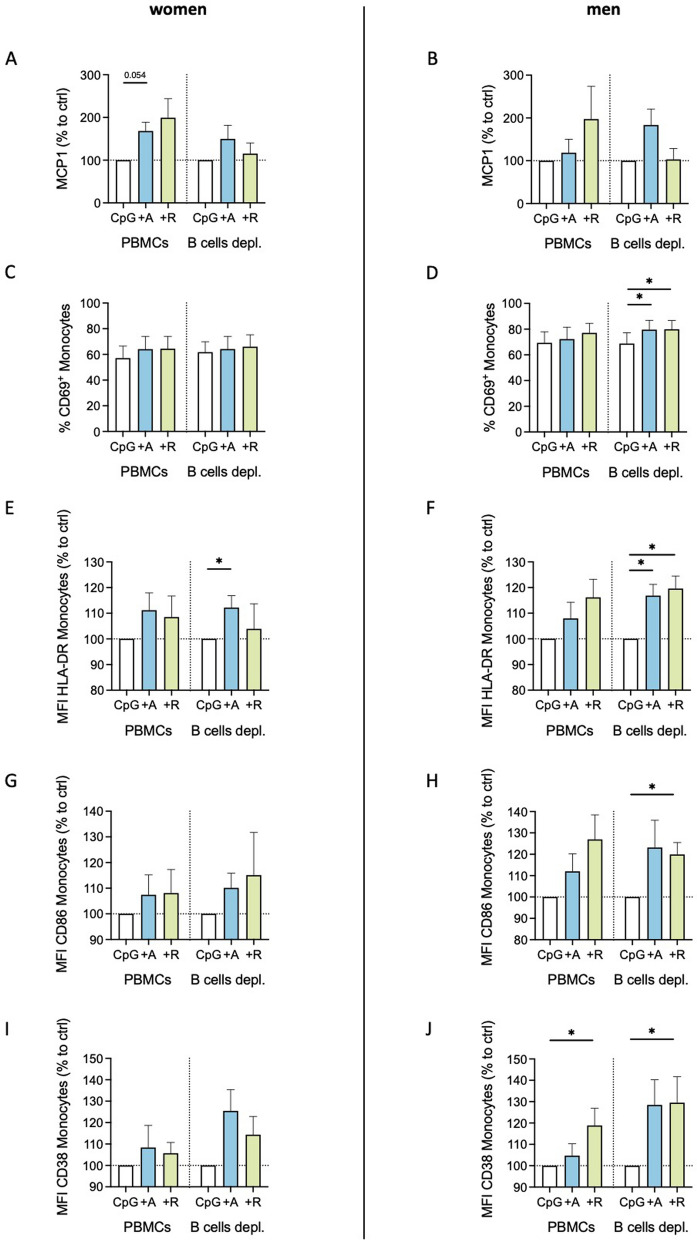
Proinflammatory phenotype of monocytes from women after DR stimulation is independent of B cells under inflammatory condition. **A**, **B** MCP1 levels in supernatant from mixed and B cell-depleted PBMCs from women (**A**) and men (**B**) after stimulation with CpG (0.195 μM), CpG + A68930 (A, 10^–7^ M) or CpG + Ropinirole (R, 10^–6^ M) measured via ELISA; normalized to CpG control; n = 7–8 per group. **C**, **D** Percentage of CD69^+^ monocytes from women (**C**) and men (**D**) after stimulation with CpG (0.195 μM), CpG + A68930 (A, 10^–7^ M) or CpG + Ropinirole (R, 10^–6^ M) measured via flow cytometry; normalized to CpG control; n = 7 per group. **E**–**J** HLA-DR (**E**, **F**), CD86 (**G**, **H**), and CD38 (**I**, **J**) expression on monocytes from women (**E**, **G**, **I**) and men (**F**, **H**, **J**) after stimulation with CpG (0.195 μM), CpG + A68930 (A, 10^–7^ M) or CpG + Ropinirole (R, 10^–6^ M) measured via flow cytometry; normalized to CpG control; n = 7 per group. Wilcoxon test was used for paired data comparisons including unstimulated vs. stimulated samples as well as PBMCs vs. B cells depleted; *p ≤ 0.05

To investigate whether the influences of dopaminergic agonists on monocyte activation and cytokine production are still driven by B cells also under inflammatory conditions, we depleted the B cells from the mixed PBMCs and analyzed MCP1 and activation marker expression after dopaminergic stimulation. Interestingly, the upregulation of MCP1 for women following DR stimulation was not observed after B cells depletion (Fig. 6A). However, the upregulation of activation markers in women and men seemed to be B cell-independent (Fig. 6C–J). Thus, we hypothesized that the B cells do slightly support the upregulation in MCP1 by monocytes under inflammatory conditions, but that DR stimulation also has a direct effect in CpG-activated monocytes leading to an even more pronounced proinflammatory phenotype.

**Fig. 7:**
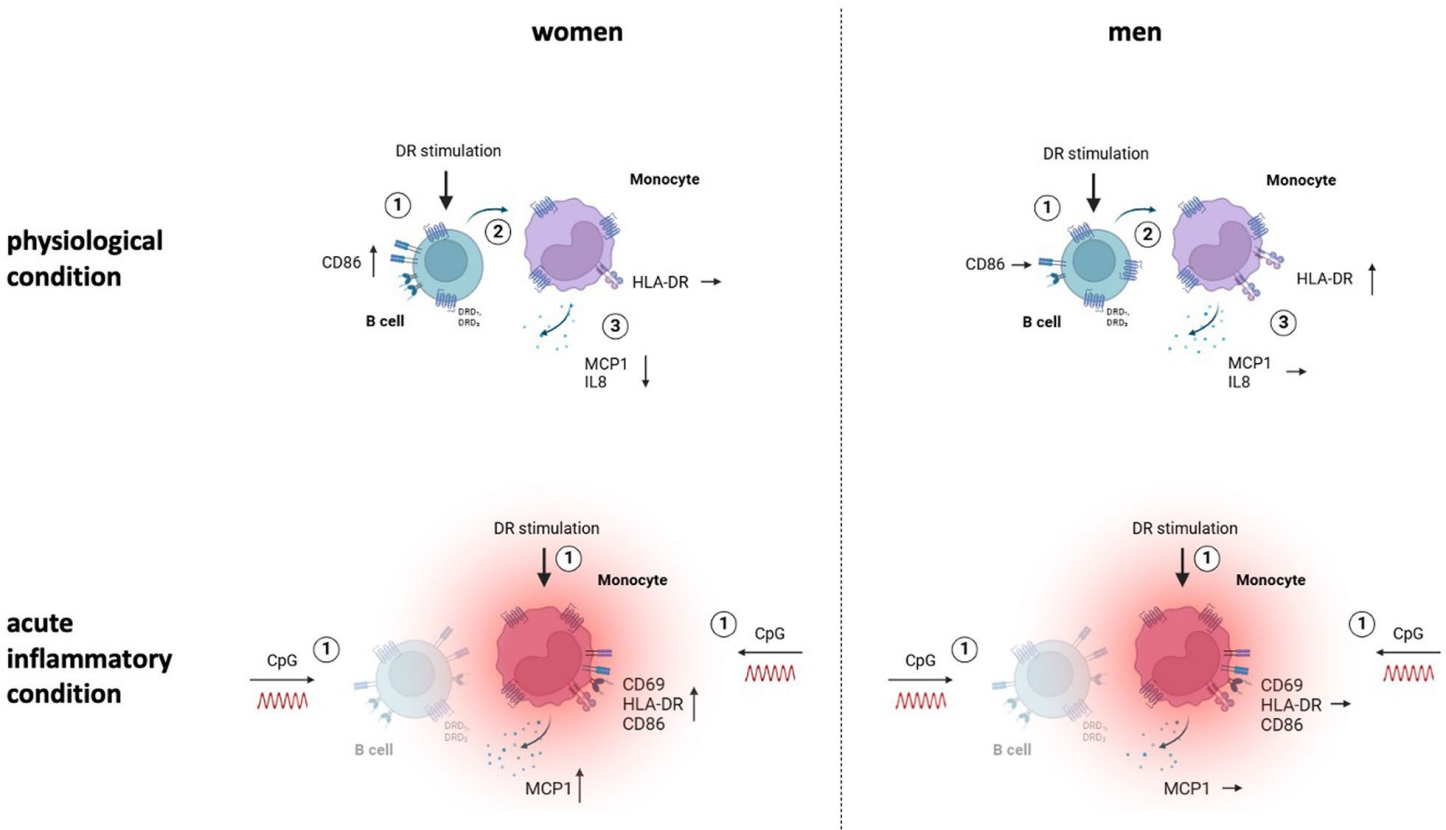
Effects of DR stimulation on female and male monocytes under physiological and acute inflammatory condition. DR stimulation dampens cytokine secretion of monocytes under physiological conditions (top), dependent on activated B cells. In men, this effect could not be observed. In acute inflammatory conditions induced by CpG (bottom), DR stimulation induces a switch into a proinflammatory phenotype of monocytes from women but not from men, independent of B cells; created in BioRender.com

Our findings about the effects of DR stimulation on PBMCs from women and men under physiological and acute inflammatory conditions are summarized in Fig. 7. For women, DR stimulation of B cells leads to an upregulation of the surface marker CD86 under healthy conditions (Step 1). Following this activation, B cells affect monocytes (Step 2), which results in the downregulation of the proinflammatory cytokines MCP1 and IL8, thus leading to an anti-inflammatory response (Step 3). Contrary for men, CD86 on B cells is not affected by DR stimulation, correlating with no downregulation of MCP1 and IL8. These effects highlight sex-specific differences in B cell-mediated immune modulation after DR stimulation. Under acute inflammatory conditions induced by CpG, which was shown to activate the monocytes directly, the effects of DR stimulation shift to a more proinflammatory phenotype only in women shown by an increased expression of activation markers on monocytes and a higher release of monocyte-derived MCP1. This switch from anti- to proinflammatory effects induced by DR stimulation is less pronounced in men. Concluding, this illustration shows the complexity of immune responses induced by DR stimulation, revealing a dependency of cell-type, activation status and sex.

## Discussion

Dopamine (DA) is a crucial bioactive compound in the human body, essential not only for physiological maintenance of various systems but also as a significant player in numerous diseases, including those involving the immune system [1, 3, 7, 34–36, 40, 41]. Within the medical field, sex-specific differences have attracted increasing attention in recent years. Given the limited understanding of DA’s effects on peripheral immune cells, it is of high interest to further investigate this link, especially considering sex as an important influencing factor. This study presents DA as a complex immunomodulator, whose effects depend on many factors such as sex and activation status of the immune cells. Moreover, DA could have indirect effects on some immune cells via activating other cell types. These complex effects are discussed in the following sections.

First of all, DA-induced effects are not necessarily DR-specific but can also occur via binding of other receptors, such as adrenoceptors [25, 47]. To prevent potential mixed effects resulting from the involvement of other pathways, we used the DR agonists A68930 and Ropinirole that specifically bind either D_1_-like or D_2_-like DRs. Besides high specificity, these agonists also offer improved stability in cell culture compared to natural DA. However, it is to be mentioned that synthetic DR-specific compounds do not fully replicate the effects that would be observed with DA in vivo, but they represent possible effects of dopaminergic drugs in systemic immune cells. Further research is needed to apply these findings to the whole human body. Of note, species-species differences in CpG sequence recognition [48] and DA’s effects on immune cells have been reported, making it challenging to directly translate results from mice to humans. For this reason, we began with in vitro experiments using mixed human PBMCs to ensure that our findings are more relevant to human physiology before proceeding to in vivo studies.

In the CNS, D_1_-like receptor binding is described to stimulate cAMP-involved pathways, while D_2_-like receptor signaling inhibits these pathways [8, 9]. However, studies on dopaminergic pathways in immune cells report diverse and sometimes contradictory results [14, 49]. The findings of Zhao et al. support the dichotomy described for the brain also in human NK cells, which show an increase in cAMP level and NK cell cytotoxicity by D_1_-like stimulation, while the opposite was observed for D_2_-like stimulation [50]. Notably, in our and other [19] studies, no opposite effects after D_1_- and D_2_-like stimulation in the modulation of immune cell functions were found, suggesting that the typical classification into the two types of receptors regarding their effects may not apply to all immune cells. Also, the exact intracellular pathways activated by DR binding in B cells and monocytes in our study remain to be elucidated. The observed sex-specific differences obtained under physiological conditions may be due to differently addressed pathways, another genetic background between women and men, or different epigenetic regulations.

In animals, it has been shown that DA’s mode of action and DR expression in the CNS can vary significantly depending on the sex [30–33]. In our study, we focused on these sex-specific differences in the periphery and found increased DRD_1_ and DRD_3_ expression in B cells from men compared to women (Fig. 2), which aligns with the findings of Wieber et al. and in vivo observations by Lévesque et al. [3, 31]. To elucidate the underlying causes of these sex-specific differences, we investigated the role of sex hormones and found an increase in DRD_1_ on B cells after E2 and DHT treatment only for men (Fig. 2). Monocytes and other PBMC subsets did not show this upregulation (data not shown), suggesting that the mechanism is not conserved across all PBMCs but specific for B cells. A previous study has described the presence of the estrogen responsive element on the promoter of DRD_1_ [33], indicating a potential direct genomic interaction. However, a direct in vivo correlation between DRD_1_ expression on B cells and sex hormone levels could not be reliably confirmed due to potential outliers and high donor-dependent variability. This variability is likely attributable to additional factors in vivo that influence DRD_1_ expression on B cells.

In this study, we observed differences in vitro between women and men not only in DR expression but also in functional assays, especially in the cytokine secretion of monocytes. While women showed a downregulation under physiological conditions, male monocytes tended to secrete more IL8 and MCP1 after physiological DR stimulation (Fig. 1). Some studies have already focused on the effect of DA on cytokine secretion, but without considering differences between sexes. For human monocyte-derived macrophages, it has been reported that DA increases the secretion of IL6 and MCP1 with and without LPS stimulation [24], while it dampened the increase in cytokine production by PBMCs under LPS-induced inflammatory conditions in another study [23]. Additionally, Hasko et al. reported an inhibition in LPS-induced IL12 p40 production of macrophages by DA, which was, however, mediated via β-adrenoceptors and not DRs [25]. Overall, the mentioned studies show very diverse contexts, and thus, their findings are only partially comparable to those in our study since we investigated the effect of DA on monocytes within mixed PBMCs. A correlation between our findings and age has not been found (data not shown).

We found that the sex-specific effects on cytokine secretion were secondarily influenced by B cells under physiological conditions. These results could be explained by the observation that B cells from women showed an upregulation in CD86, increasing their activation and antigen-presenting capabilities [39, 51], which was not observed for B cells from men (Fig. 3). This suggests a change in the interaction with monocytes, leading to a suppression of monocytes and thus reduced cytokine secretion. The precise mechanism of this interaction in our study still needs to be investigated. In this context, it is described that B cells and monocytes can interact either via direct cell–cell contact [52] or via soluble factors like B cell-derived cytokines such as IL10 or lymphotoxin, modulating the functions of monocytes [53, 54]. In summary, coculture with PBMCs was essential to find out the indirect effects of DA on monocytes via B cells. Of note, inflammasome activation could also cause the observed effects. This is especially supported by the increase of IL1β and IL18 secretion by female PBMCs. While CpG is not described to induce inflammasome formation, a relation between dopaminergic stimulation and a priming and/or activation of the NLRP3 inflammasome is reported in a few studies [55, 56].

Besides the influencing factors of sex and secondary impacts by surrounding immune cells, a key player in modulating DA’s effects is the activation status of the cells. In line with this, it has been reported that the effects of DR stimulation vary when other receptors or signaling pathways are coactivated [45]. In our study, the reduced cytokine secretion of monocytes from women switched to an upregulation when the TLR9 pathway was coactivated via CpG stimulation (Supplementary Fig. 7). The MAPK pathway was reported to alter the effects of DA [45], and is also activated by TLR9 signaling [46], supporting the hypothesis that coactivated pathways can influence the outcomes of DR signaling. Whether this switch is indeed due to MAPK involvement in female monocytes after CpG stimulation requires further investigation. Unfortunately, studying the direct effect of DR stimulation on isolated monocytes is challenging, since monocytes died under comparable cell culture conditions within a few hours after thawing and isolation (data not shown) probably due to high stress and missing signals from other PBMCs.

Taken together, our results show how DR stimulation affects peripheral immune cells in a sex-specific manner and thus importantly contributes to the deepened understanding of DA’s effects on the immune system in both women and men. For instance, DA may reduce unwarranted immune responses in the healthy peripheral immune system only in women, potentially serving as a protective mechanism against autoimmune diseases. Conversely, under acute inflammatory conditions, such as bacterial infections, DA may enhance proinflammatory effects to clear pathogens but could also enhance autoreactive responses.

Several questions remain to be addressed in further studies. Firstly, the effects of dopaminergic stimulation on B cells should be examined in further research, to elucidate their interaction with monocytes and ability to influence their cytokine secretion in a sex-specific manner. Exploring whether this effect is stimulus-specific or whether similar effects can be obtained by stimulation with other activating substances like LPS could further deepen our knowledge about the working mechanism of DA. Moreover, other PBMC subtypes might be influenced by or have an influence on the effects we observed, though addressing this complexity is challenging. We observed increased activation of T cells, shown by increased HLA-DR and CD86 expression, independent of an applied stimulus (Supplementary Fig. 8), likely due to direct DR stimulation on T cells as described in several studies. These effects on T cells were independent of sex and show that the differences between women and men observed for monocytes and B cells are cell specific. Also, further experiments including dopamine and further agonists with specific antagonists could clarify the pharmacological signal activated by DA in immune cells.

## Conclusion

In summary, our study demonstrates that dopaminergic stimulation induces sex-specific effects on PBMCs from women and men. Under physiological conditions, we observed B cell-driven anti-inflammatory effects on monocytes, shown in reduced cytokine secretion and activation marker expression exclusively in women. These sex-specific differences are accompanied by increased DR expression on B cells in men, which could further be increased by treatment with sex hormones, which supports their influencing factors in the interplay of the dopaminergic pathway and immune cell function. Interestingly, our findings showed that coactivation of the TLR9 pathway via CpG, mimicking an acute inflammatory stimulus, led to a switch from anti-inflammatory to proinflammatory responses in women after DR stimulation. These findings improve our understanding of DA’s mode of action under both healthy and inflammatory conditions in women and men, highlighting sex as an influencing factor in dopaminergic effects on PBMCs. They offer valuable insights for developing targeted therapeutic strategies for DA-related pathologies and emphasize the importance of considering sex-specific treatments. Further research is needed to elucidate its effects in vivo.

## Supplementary Information

Supplementary material 1: Figure 1. Sex-specific effects of DR stimulation on immunological responses of monocytes under physiological conditions. A Flow chart of experiments performed. *PBMC* peripheral blood mononuclear cells, *DR* dopamine receptor, *SHR* sex hormone receptor, *ER* estrogen receptor, *AR* androgen receptor. (Created in https://BioRender.com) B Flow cytometry gating strategy for mixed PBMCs and representative histograms for DRD_1_, DRD_2_, DRD_3_, DRD_4_, CD69, HLA-DR, CD86, and CD38 expression on monocytes. This gating strategy was consistently applied across all flow cytometry experiments conducted in this study. Figure 2. Sex-specific effects of DR stimulation on immunological responses of monocytes under physiological conditions. A, B Basal IL8 (A) and MCP1 (B) levels in supernatants from PBMCs of women and men after 24 h in cell culture without *in vitro *stimulation measured via ELISA; n=11-13 per group. C, D IL8 (C) and MCP1 (D) levels in supernatants from PBMCs of women and men after 24 h in cell culture, with and without *in vitro *stimulation by A68930 (A, 10^-7^, 10^-8^, 10^-9^ M) or Ropinirole (R, 10^-6^, 10^-7^, 10^-8^ M) measured via ELISA; normalized to unstimulated control; data of A 10^-7^ M and R 10^-6^ M are the same as in Fig. 1 A-D; n=11-13 per group. E, F Percentage of IL8^+^ (E) and MCP1^+^ (F) B cells, monocytes, T cells, and NK cells after 24 h in culture without stimulation measured via flow cytometry; n=5 per subtype. G, H, I, K Percentage of CD69^+^ monocytes (G) and basal expression level of HLA-DR (H), CD86 (I) and CD38 (K) on monocytes from women and men after 24 h in culture of mixed PBMCs after 24 h in cell culture without *in vitro *stimulation measured via flow cytometry; n=13-14 per group. J, L CD86 (J) and CD38 (L) expression on monocytes from women and men after 24 h in culture of mixed PBMCs with or without stimulation by A68930 (A, 10^-7^ M) or Ropinirole (R, 10^-6^ M) measured via flow cytometry; normalized to unstimulated control; n=13-14 per group. One-way ANOVA or mixed-effects analysis with Geisser-Greenhouse correction and Dunnett multiple comparisons test was used for statistical testing of DR stimulation using three concentrations of A68930 and Ropinirole. Mann-Whitney test was used for testing statistical significance between unpaired data of women and men. Wilcoxon test was used for comparison of paired data including unstimulated vs. stimulated samples; *p ≤ 0.05. Figure 3. Women and men exhibit similar estrogen levels, while testosterone is higher in men. A–D Basal expression of ERα (A), ERβ (B), GPR30 (C) and AR (D) on B cells from women and men measured via flow cytometry; n=6 per group. E, G Basal estrogen (E) and testosterone (G) levels in plasma from women and men measured via ELISA; n=17-19 per group. F Basal estrogen levels in plasma from women with (w, circles) and without (w/o, squares) hormonal contraception measured via ELISA; n=5-14 per group. Mann-Whitney test was used for testing statistical significance between data of women and men or women with and without hormonal contraception; *p ≤ 0.05, ****p ≤ 0.0001. Figure 4. Effects of DR stimulation on B cells from women and men. A–C Basal expression level of CD86 (A), CD69 (B) and HLA-DR (C) on B cells from PBMCs of women and men after 24 h in cell culture without *in vitro *stimulation measured via flow cytometry; n=13-14 per group. D, E CD69 (D) and HLA-DR (E) expression on B cells from women and men after 24 h in mixed PBMC culture, with or without stimulation by A68930 (A, 10^-7^ M) and Ropinirole (R, 10^-6^ M) measured via flow cytometry; normalized to unstimulated control; n=13-14 per group. Mann-Whitney test was used for testing statistical significance between unpaired data of women and men. Wilcoxon test was used for comparison of paired data including unstimulated vs. stimulated samples; *p ≤ 0.05, **p ≤ 0.01. Figure 5. Basal levels of cytokines and activation marker expression on monocytes without and with CpG stimulation. A Percentage of CD69^+^ B cells after 24 h in mixed PBMC culture with or without CpG (0.195 μM) stimulation measured via flow cytometry; n=30 per condition. B Percentage of CD69^+^ monocytes after 24 h in culture of mixed and B cell-depleted PBMCs with or without CpG (0.195 μM) stimulation measured via flow cytometry; n=14 per condition. C MCP1 levels in supernatant from PBMCs of women and men after 24 h in culture, with or without CpG (0.195 μM) stimulation measured via ELISA; n=11-13 per group. D–G Percentage of CD69^+^ monocytes (D) and expression of HLA-DR (E), CD86 (F) and CD38 (G) on monocytes from women and men after 24 h in culture of PBMCs with or without CpG (0.195 μM) stimulation; n=14 per group. Wilcoxon test was used for paired data comparisons including unstimulated vs. stimulated samples as well as PBMCs vs. B cells depleted. Mann-Whitney test was used for testing statistical significance between unpaired data of women and men; *p ≤ 0.05, **p ≤ 0.01, ***p ≤ 0.001, ****p ≤ 0.0001. Figure 6. Effect of DR stimulation on secretion of other cytokines of mixed PBMCs. A Percentage of IL8^+^ B cells, monocytes, T cells, and NK cells after 24 h in PBMC culture, with or without CpG (0.195 μM) stimulation measured via flow cytometry; n=12 per group. B IL8 levels in supernatant from mixed and monocyte-depleted PBMCs after 24 h of CpG (0.195 μM) stimulation measured via ELISA; n=11 per condition. C, D IL8 levels in supernatant from PBMCs of women and men after 24 h in culture with or without CpG (0.195 μM) stimulation, and after stimulation with CpG (0.195 μM) + A68930 (A, 10^-7^ M) or CpG (0.195 μM) + Ropinirole (R, 10^-6^ M) measured via ELISA; D was normalized to CpG control; n=11-13 per group. E Percentage of MCP1^+^ B cells, T cells, and NK cells after stimulation of PBMCs with CpG (0.195 μM) + A68930 (A, 10^-7^ M) or CpG (0.195 μM) + Ropinirole (R, 10^-6^ M) for 24 h measured via flow cytometry; n=6 per group. F, G MCP1 levels in supernatant from mixed PBMCs from women (F) and men (G) after stimulation with A68930 (A, 10^-7^ M), Ropinirole (R, 10^-6^ M), CpG (0.195 μM) + A68930 (A, 10^-7^ M) or CpG (0.195 μM) + Ropinirole (R, 10^-6^ M) for 24 h measured via ELISA; normalized to unstimulated or CpG control, respectively; n=11 per condition. H, I IL1β (H) and IL18 (I) levels in supernatant from mixed and monocyte-depleted PBMCs after 24 h of CpG (0.195 μM) stimulation measured via Legendplex; n=9-11 per condition. J, K IL1β (J) and IL18 (K) levels in supernatant from PBMCs of women and men after 24 h in culture with or without CpG (0.195 μM) stimulation measured via Legendplex, n=9-10 per group. L, M IL1β (L) and IL18 (M) levels in supernatant from mixed PBMCs of women and men after stimulation with CpG (0.195 μM), CpG (0.195 μM) + A68930 (A, 10^-7^ M) or CpG (0.195 μM) + Ropinirole (R, 10^-6^ M) for 24 h measured via Legendplex; normalized to CpG control; n=9-10 per group. Wilcoxon test was used for comparison of paired data including unstimulated vs. stimulated samples as well as PBMCs vs. monocytes depleted. Mann-Whitney test was used for testing statistical significance between unpaired data of women and men; *p ≤ 0.05, **p ≤ 0.01, ***p ≤ 0.001. Figure 7. Comparison of effects on activation marker expression of monocytes after DR stimulation with and without an inflammatory stimulus. A–H Percentage of CD69^+^ monocytes (A, B) and expression of HLA-DR (C, D), CD86 (E, F) and CD38 (G, H) on monocytes from women (A, C, E, G) and men (B, D, F, H) after stimulation of PBMCs with A68930 (A, 10^-7^ M), Ropinirole (R, 10^-6^ M), CpG (0.195 μM) + A68930 (A, 10^-7^ M) and CpG (0.195 μM) + Ropinirole (R, 10^-6^ M) for 24 h measured via flow cytometry; normalized to unstimulated or CpG control, respectively; n=14 per condition. Wilcoxon test was used for comparison of paired data including unstimulated vs. stimulated samples; **p ≤ 0.01, ***p ≤ 0.001. Figure 8. Increase in HLA-DR on T cells after DR stimulation is independent of sex and inflammation. A–D HLA-DR (A, B) and CD86 (C, D) expression on T cells from women and men after 24 h in mixed PBMC culture with or without stimulation by A68930 (A, 10^-7^ M) or Ropinirole (R, 10^-6^ M) measured via flow cytometry; B and D were normalized to unstimulated control; n=13-14 per group. E–H HLA-DR (E, F) and CD86 (G, H) expression on T cells from women and men after 24 h in mixed PBMC culture with or without CpG (0.195 μM) stimulation, and after stimulation with CpG (0.195 μM) + A68930 (A, 10^-7^ M) or CpG + Ropinirole (R, 10^-6^ M) measured via flow cytometry; F and H were normalized to CpG control; n=11-12 per group. Mann-Whitney test was used for testing statistical significance between unpaired data of women and men. Wilcoxon test was used for comparison of paired data including unstimulated vs. stimulated samples; *p ≤ 0.05, **p ≤ 0.01, ***p ≤ 0.001. Table 1. Antibodies used for flow cytometry staining of dopamine receptors, sex hormone receptors and activation markers including Annexin V. Table 2. Antibodies used for Intracellular cytokine measurement via flow cytometry.

## Data Availability

The datasets supporting the conclusions of this article are included within the article and its additional file(s). On reasonable request, the corresponding author will provide access to the data used and/or analyzed in this study.

## Abbreviations

DA: Dopamine
CNS: Central nervous system
DR: Dopamine receptor
cAMP: Cyclic adenosine monophosphate
PBMCs: Peripheral blood mononuclear cells
LPS: Lipopolysaccharide
SLE: Systemic lupus erythematosus
RA: Rheumatoid arthritis
TLR9: Toll-like receptor 9
PBS: Phosphate-buffered saline
FITC: Fluorescein isothiocyanate
FACS: Fluorescence activated cell sorting
IL: Interleukin
MCP1: Monocyte chemotactic protein 1
CD: Cluster of differentiation
HLA-DR: Human Leukocyte Antigen DR isotype
ER: Estrogen receptor
AR: Androgen receptor
GPR30: G protein-coupled receptor 30
E2: 17β-Estradiol
DHT: Dihydrotestosterone
SHR: Sex hormone receptor
MZ: Marginal zone-like
SM: Switched memory
