# Supplementary material for: Acute stimulation of PBMCs drives switch from dopamine-induced anti- to proinflammatory phenotype of monocytes only in women
Source: Biol Sex Differ. 2025 Feb 3;16:8. doi: 10.1186/s13293-025-00689-5 (PMC11789415; doi:10.1186/s13293-025-00689-5)
Supplement: Supplementary file 1 — Supplementary material 1: Figure 1. Sex-specific effects of DR stimulation on immunological responses of monocytes under physiological conditions. A Flow chart of experiments performed. PBMC peripheral blood mononuclear cells, DR dopamine receptor, SHR sex hormone receptor, ER estrogen receptor, AR androgen receptor. (Created in https://BioRender.com) B Flow cytometry gating strategy for mixed PBMCs and representative histograms for DRD1, DRD2, DRD3, DRD4, CD69, HLA-DR, CD86, and CD38 expression on monocytes. This gating strategy was consistently applied across all flow cytometry experiments conducted in this study. Figure 2. Sex-specific effects of DR stimulation on immunological responses of monocytes under physiological conditions. A, B Basal IL8 (A) and MCP1 (B) levels in supernatants from PBMCs of women and men after 24 h in cell culture without in vitro stimulation measured via ELISA; n=11-13 per group. C, D IL8 (C) and MCP1 (D) levels in supernatants from PBMCs of women and men after 24 h in cell culture, with and without in vitro stimulation by A68930 (A, 10-7, 10-8, 10-9 M) or Ropinirole (R, 10-6, 10-7, 10-8 M) measured via ELISA; normalized to unstimulated control; data of A 10-7 M and R 10-6 M are the same as in Fig. 1 A-D; n=11-13 per group. E, F Percentage of IL8+ (E) and MCP1+ (F) B cells, monocytes, T cells, and NK cells after 24 h in culture without stimulation measured via flow cytometry; n=5 per subtype. G, H, I, K Percentage of CD69+ monocytes (G) and basal expression level of HLA-DR (H), CD86 (I) and CD38 (K) on monocytes from women and men after 24 h in culture of mixed PBMCs after 24 h in cell culture without in vitro stimulation measured via flow cytometry; n=13-14 per group. J, L CD86 (J) and CD38 (L) expression on monocytes from women and men after 24 h in culture of mixed PBMCs with or without stimulation by A68930 (A, 10-7 M) or Ropinirole (R, 10-6 M) measured via flow cytometry; normalized to unstimulated control; n=13-14 per group. One-w [file 13293_2025_689_MOESM1_ESM.pdf]

Figures:
Acute stimulation of PBMCs drives switch from dopamine-induced anti- to proinflammatory phenotype of monocytes only in women

Leonie Fleige[1], Silvia Capellino[1*]

[1]Department of Immunology, Research Group of Neuroimmunology, IfADo-Leibniz Research Centre for Working Environment and Human Factors, Ardeystraße 67, 44139 Dortmund, Germany. LF: fleige@ifado.de; SC: capellino@ifado.de

* Corresponding author. E-mail address: capellino@ifado.de.

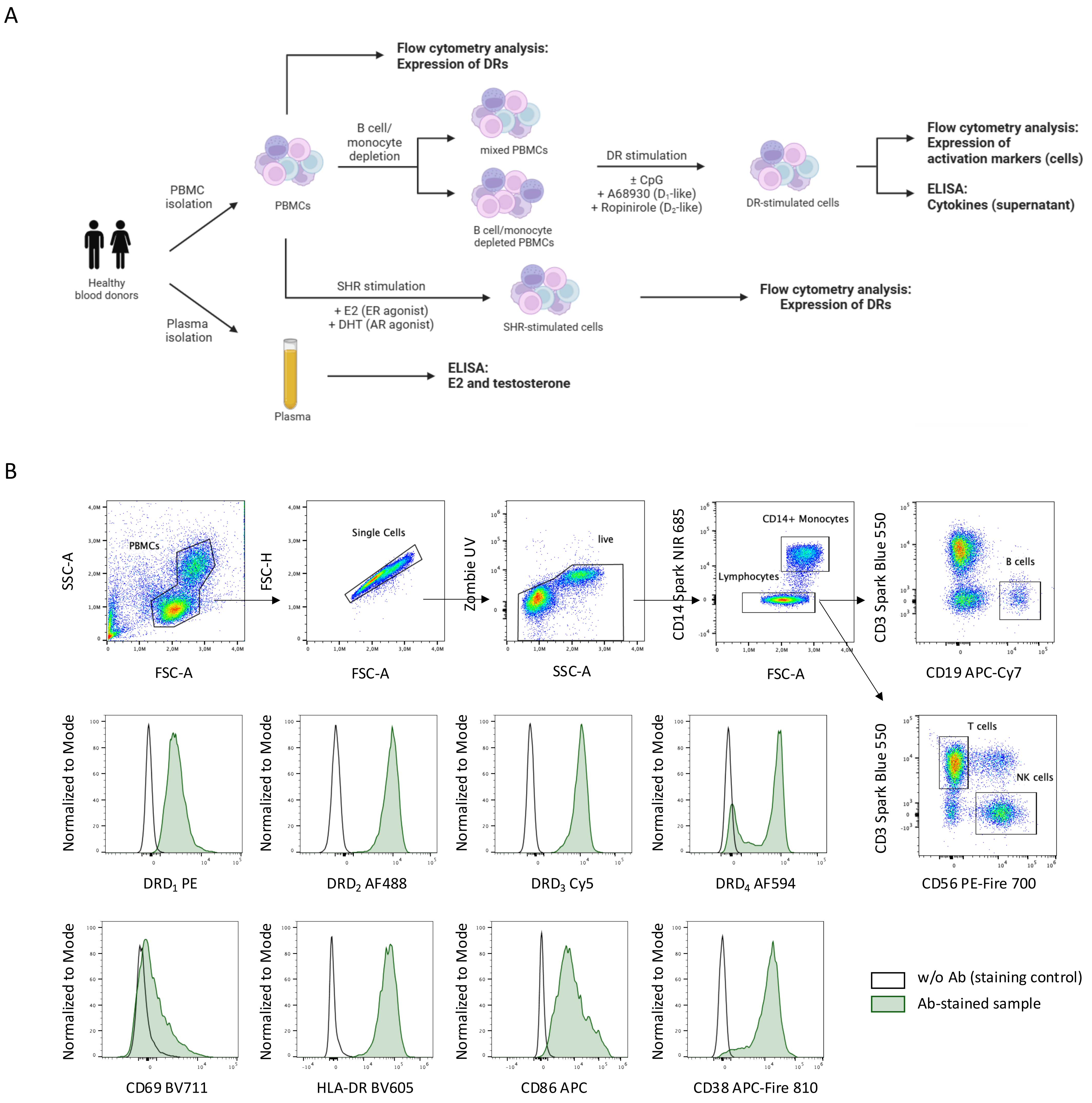

Supplementary Figure 1: Sex-specific effects of DR stimulation on immunological responses of monocytes under physiological conditions. A) Flow chart of experiments performed. PBMC: peripheral blood mononuclear cells; DR: dopamine receptor; SHR: sex hormone receptor; ER: estrogen receptor; AR: androgen receptor (Created in https://BioRender.com). B) Flow cytometry gating strategy for mixed PBMCs and representative histograms for DRD_1, DRD_2, DRD_3, DRD_4, CD69, HLA-DR, CD86, and CD38 expression on monocytes. This gating strategy was consistently applied across all flow cytometry experiments conducted in this study.

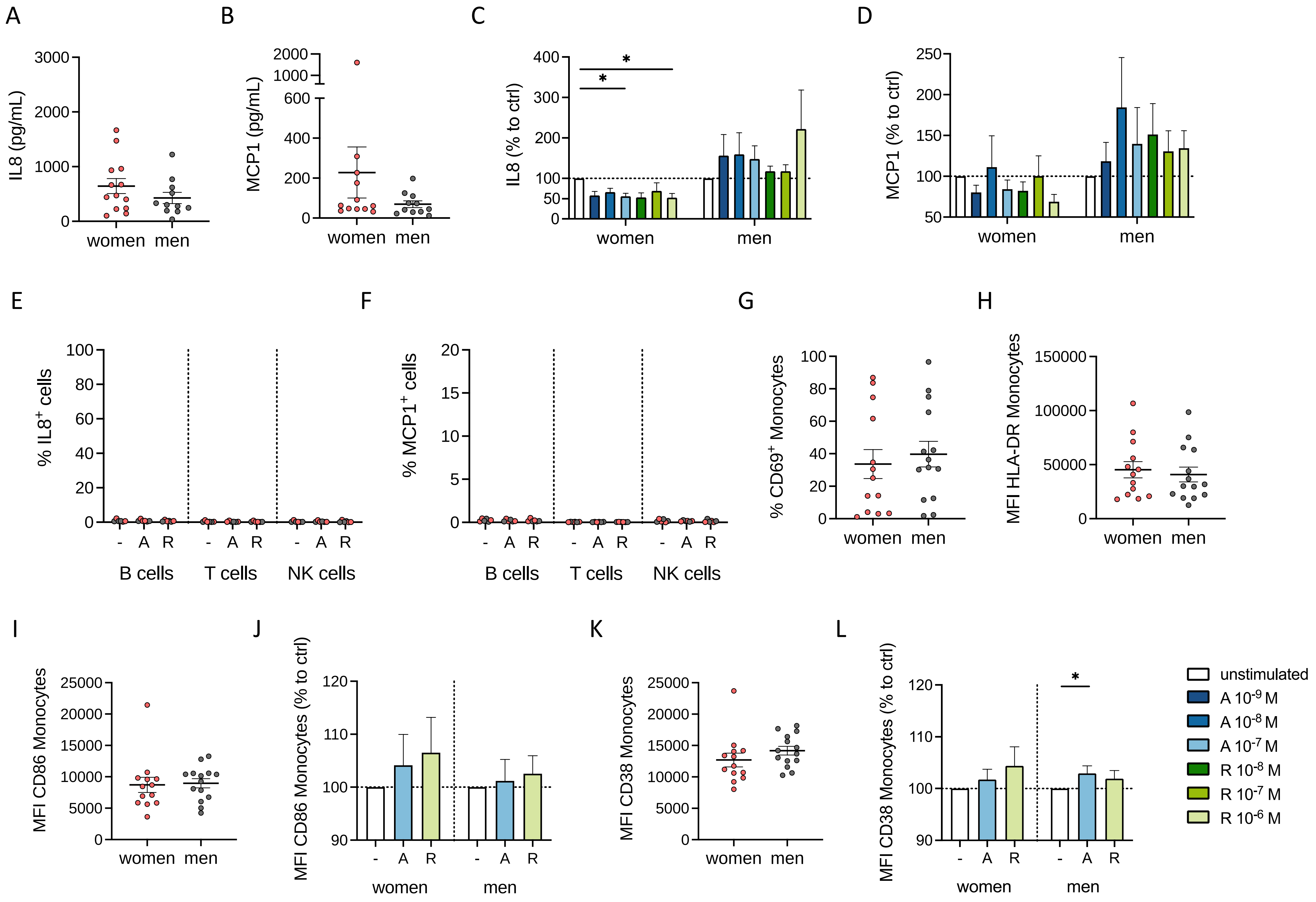

Supplementary Figure 2: Sex-specific effects of DR stimulation on immunological responses of monocytes under physiological conditions. A, B) Basal IL8 (A) and MCP1 (B) levels in supernatants from PBMCs of women and men after 24 h in cell culture without *in vitro* stimulation measured via ELISA; n=11-13 per group. **C, D**) IL8 (C) and MCP1 (D) levels in supernatants from PBMCs of women and men after 24 h in cell culture, with and without *in vitro* stimulation by A68930 (A, 10^{-7}, 10^{-8}, 10^{-9} M) or Ropinirole (R, 10^{-6}, 10^{-7}, 10^{-8} M) measured via ELISA; normalized to unstimulated control; data of A 10^{-7} M and R 10^{-6} M are the same as in Figure 1 A-D; n=11-13 per group. **E, F**) Percentage of $IL8^+$ (E) and $MCP1^+$ (F) B cells, monocytes, T cells, and NK cells after 24 h in culture without stimulation measured via flow cytometry; n=5 per subtype. **G, H, I, K**) Percentage of $CD69^+$ monocytes (G) and basal expression level of HLA-DR (H), CD86 (I) and CD38 (K) on monocytes from women and men after 24 h in culture of mixed PBMCs after 24 h in cell culture without *in vitro* stimulation measured via flow cytometry; n=13-14 per group. **J, L**) CD86 (J) and CD38 (L) expression on monocytes from women and men after 24 h in culture of mixed PBMCs with or without stimulation by A68930 (A, 10^{-7} M) or Ropinirole (R, 10^{-6} M) measured via flow cytometry; normalized to unstimulated control; n=13-14 per group. One-way ANOVA or mixed-effects analysis with Geisser-Greenhouse correction and Dunnett multiple comparisons test was used for statistical testing of DR stimulation using three concentrations of A68930 and Ropinirole. Mann-Whitney test was used for testing statistical significance between unpaired data of women and men. Wilcoxon test was used for comparison of paired data including unstimulated vs. stimulated samples; *$p \le 0.05$.

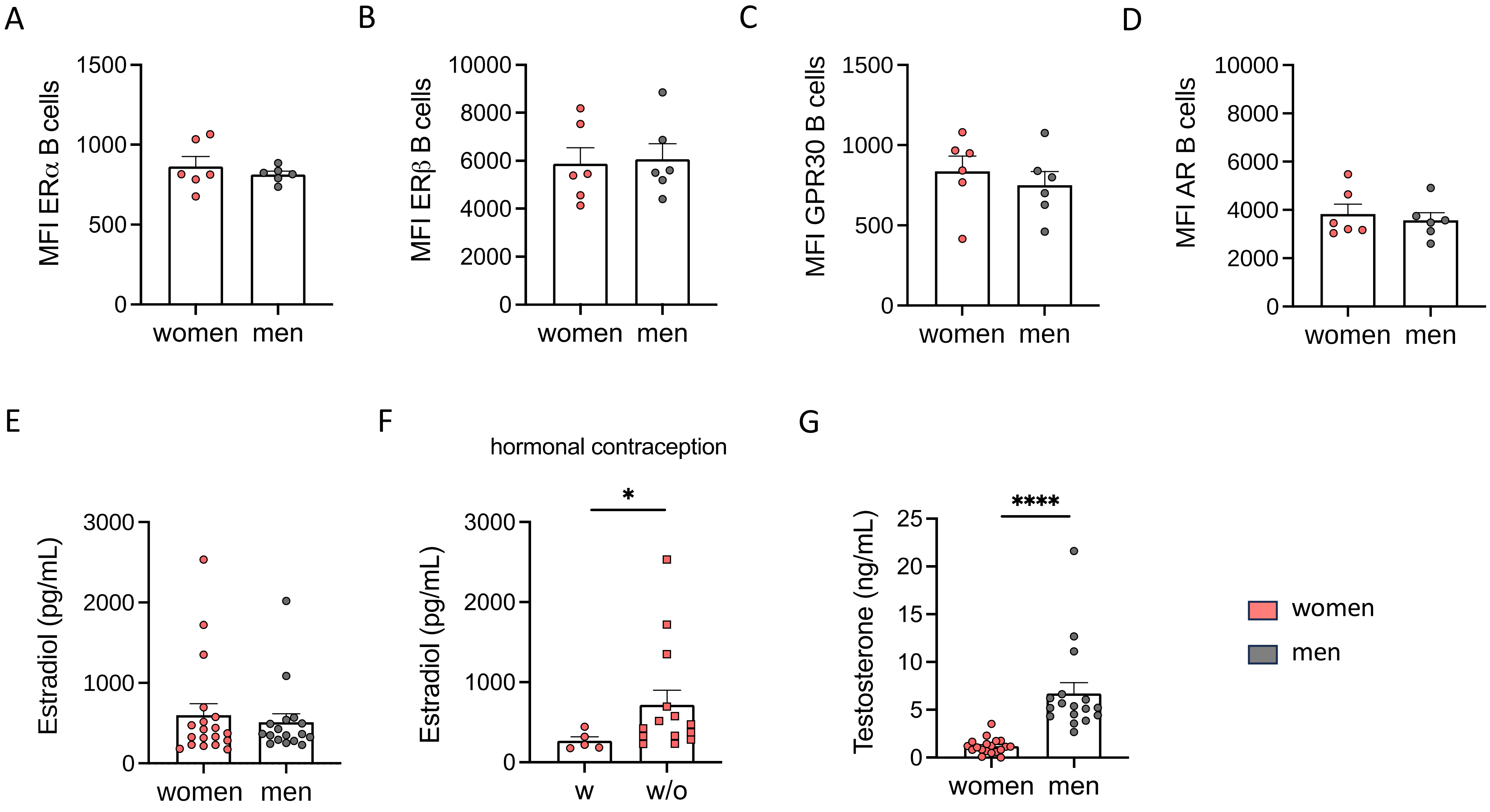

Supplementary Figure 3: Women and men exhibit similar estrogen levels, while testosterone is higher in men. A-D) Basal expression of ERα (A), ERβ (B), GPR30 (C) and AR (D) on B cells from women and men measured via flow cytometry; n=6 per group. **E, G**) Basal estrogen (E) and testosterone (G) levels in plasma from women and men measured via ELISA; n=17-19 per group. **F**) Basal estrogen levels in plasma from women with (w, circles) and without (w/o, squares) hormonal contraception measured via ELISA; n=5-14 per group. Mann-Whitney test was used for testing statistical significance between data of women and men or women with and without hormonal contraception; *p ≤ 0.05, ****p ≤ 0.0001.

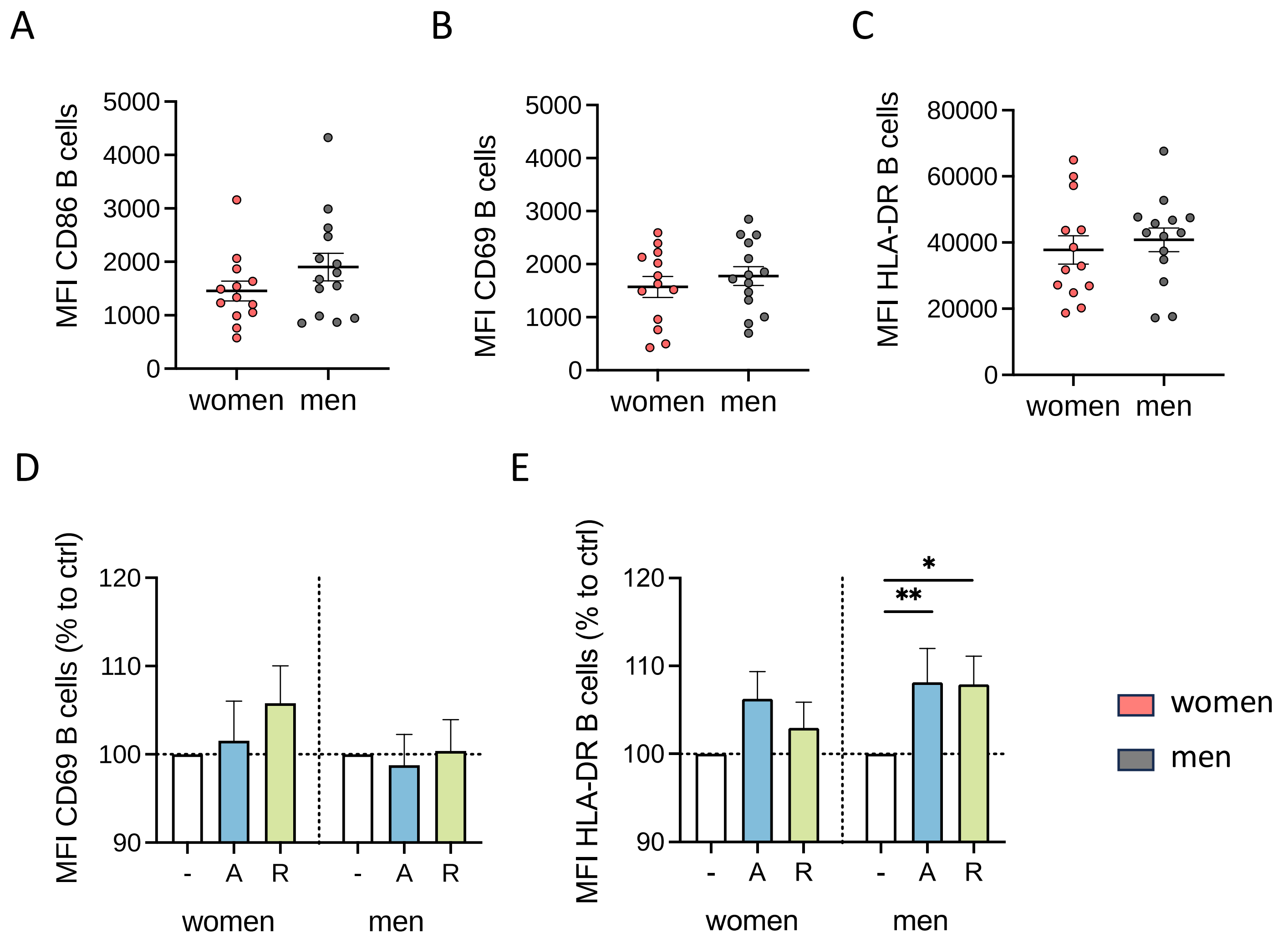

Supplementary Figure 4: Effects of DR stimulation on B cells from women and men. A-C) Basal expression level of CD86 (A), CD69 (B) and HLA-DR (C) on B cells from PBMCs of women and men after 24 h in cell culture without *in vitro* stimulation measured via flow cytometry; n=13-14 per group. **D, E**) CD69 (D) and HLA-DR (E) expression on B cells from women and men after 24 h in mixed PBMC culture, with or without stimulation by A68930 (A, 10^{-7} M) and Ropinirole (R, 10^{-6} M) measured via flow cytometry; normalized to unstimulated control; n=13-14 per group. Mann-Whitney test was used for testing statistical significance between unpaired data of women and men. Wilcoxon test was used for comparison of paired data including unstimulated vs. stimulated samples; *$p \leq 0.05$, **$p \leq 0.01$.

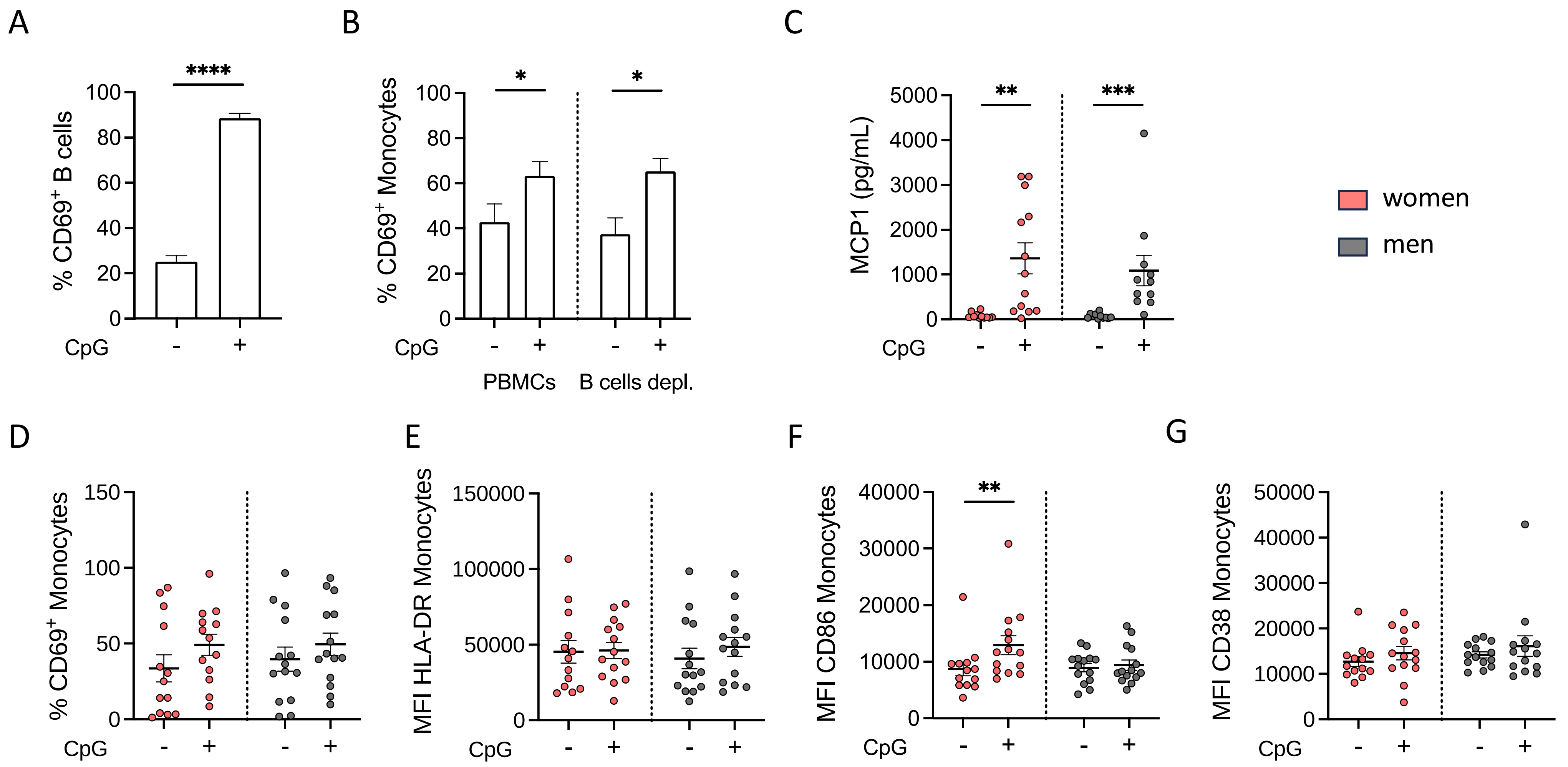

Supplementary Figure 5: Basal levels of cytokines and activation marker expression on monocytes without and with CpG stimulation. A) Percentage of $CD69^+$ B cells after 24 h in mixed PBMC culture with or without CpG (0.195 μM) stimulation measured via flow cytometry; n=30 per condition. **B**) Percentage of $CD69^+$ monocytes after 24 h in culture of mixed and B cell-depleted PBMCs with or without CpG (0.195 μM) stimulation measured via flow cytometry; n=14 per condition. **C**) MCP1 levels in supernatant from PBMCs of women and men after 24 h in culture, with or without CpG (0.195 μM) stimulation measured via ELISA; n=11-13 per group. **D-G**) Percentage of $CD69^+$ monocytes (D) and expression of HLA-DR (E), CD86 (F) and CD38 (G) on monocytes from women and men after 24 h in culture of PBMCs with or without CpG (0.195 μM) stimulation; n=14 per group. Wilcoxon test was used for paired data comparisons including unstimulated vs. stimulated samples as well as PBMCs vs. B cells depleted. Mann-Whitney test was used for testing statistical significance between unpaired data of women and men; *$p \leq 0.05$, **$p \leq 0.01$, ***$p \leq 0.001$, ****$p \leq 0.0001$.

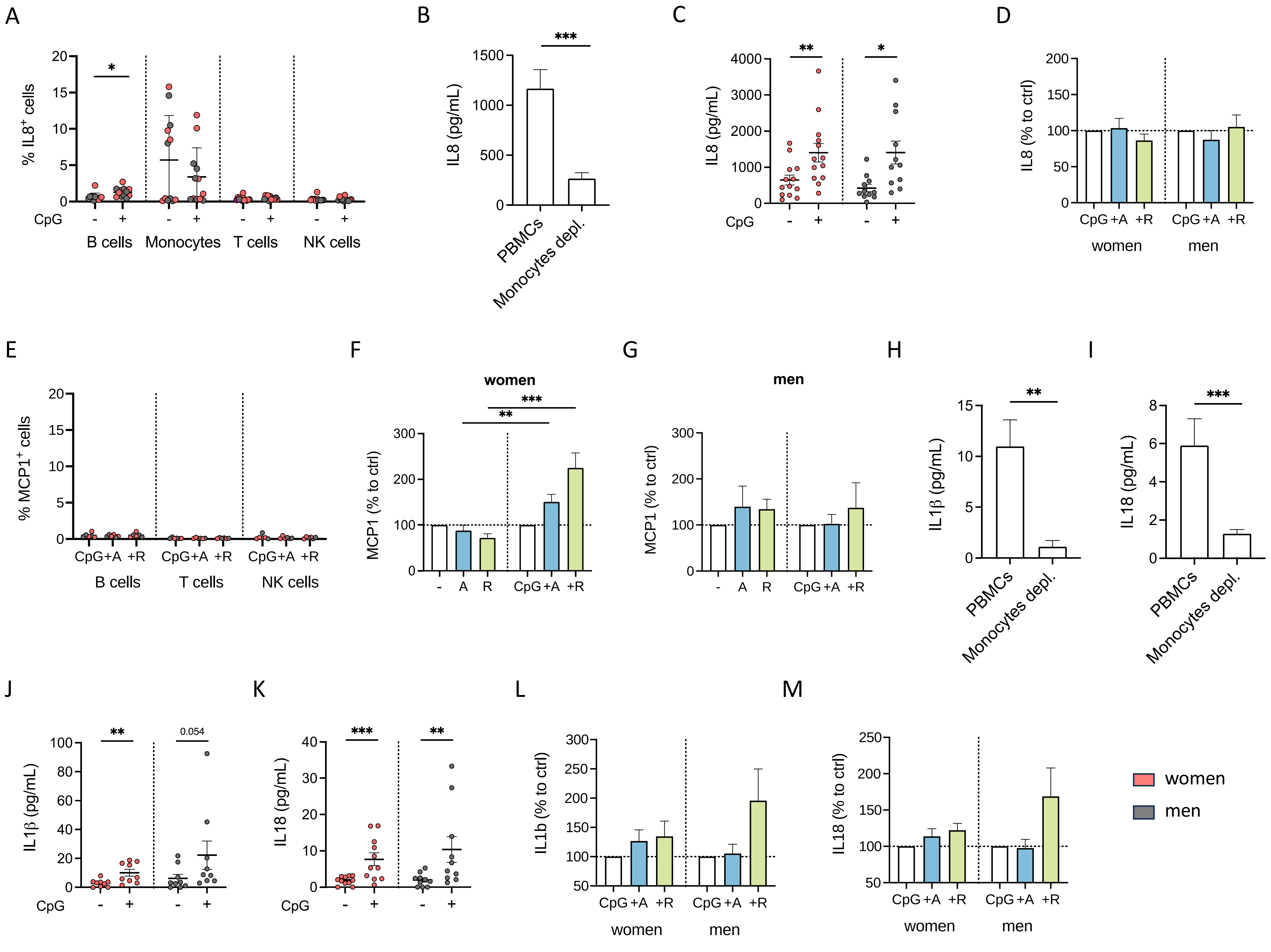

Supplementary Figure 6: Effect of DR stimulation on secretion of other cytokines of mixed PBMCs. A) Percentage of IL8⁺ B cells, monocytes, T cells, and NK cells after 24 h in PBMC culture, with or without CpG (0.195 μM) stimulation measured via flow cytometry; n=12 per group. **B**) IL8 levels in supernatant from mixed and monocyte-depleted PBMCs after 24 h of CpG (0.195 μM) stimulation measured via ELISA; n=11 per condition. **C, D**) IL8 levels in supernatant from PBMCs of women and men after 24 h in culture with or without CpG (0.195 μM) stimulation, and after stimulation with CpG (0.195 μM) + A68930 (A, 10^{-7} M) or CpG (0.195 μM) + Ropinirole (R, 10^{-6} M) measured via ELISA; D was normalized to CpG control; n=11-13 per group. **E**) Percentage of MCP1⁺ B cells, T cells, and NK cells after stimulation of PBMCs with CpG (0.195 μM) + A68930 (A, 10^{-7} M) or CpG (0.195 μM) + Ropinirole (R, 10^{-6} M) for 24 h measured via flow cytometry; n=6 per group. **F, G**) MCP1 levels in supernatant from mixed PBMCs from women (F) and men (G) after stimulation with A68930 (A, 10^{-7} M), Ropinirole (R, 10^{-6} M), CpG (0.195 μM) + A68930 (A, 10^{-7} M) or CpG (0.195 μM) + Ropinirole (R, 10^{-6} M) for 24 h measured via ELISA; normalized to unstimulated or CpG control, respectively; n=11 per condition. **H, I**) IL1β (H) and IL18 (I) levels in supernatant from mixed and monocyte-depleted PBMCs after 24 h of CpG (0.195 μM) stimulation measured via Legendplex; n=9-11 per condition. **J, K**) IL1β (J) and IL18 (K) levels in supernatant from PBMCs of women and men after 24 h in culture with or without CpG (0.195 μM) stimulation measured via Legendplex, n=9-10 per group. **L, M**) IL1β (L) and IL18 (M) levels in supernatant from mixed PBMCs of women and men after stimulation with CpG (0.195 μM), CpG (0.195 μM) + A68930 (A, 10^{-7} M) or CpG (0.195 μM) + Ropinirole (R, 10^{-6} M) for 24 h measured via Legendplex; normalized to CpG control; n=9-10 per group. Wilcoxon test was used for comparison of paired data including unstimulated vs. stimulated samples as well as PBMCs vs. monocytes depleted. Mann-Whitney test was used for testing statistical significance between unpaired data of women and men; *$p \leq 0.05$, **$p \leq 0.01$, ***$p \leq 0.001$.

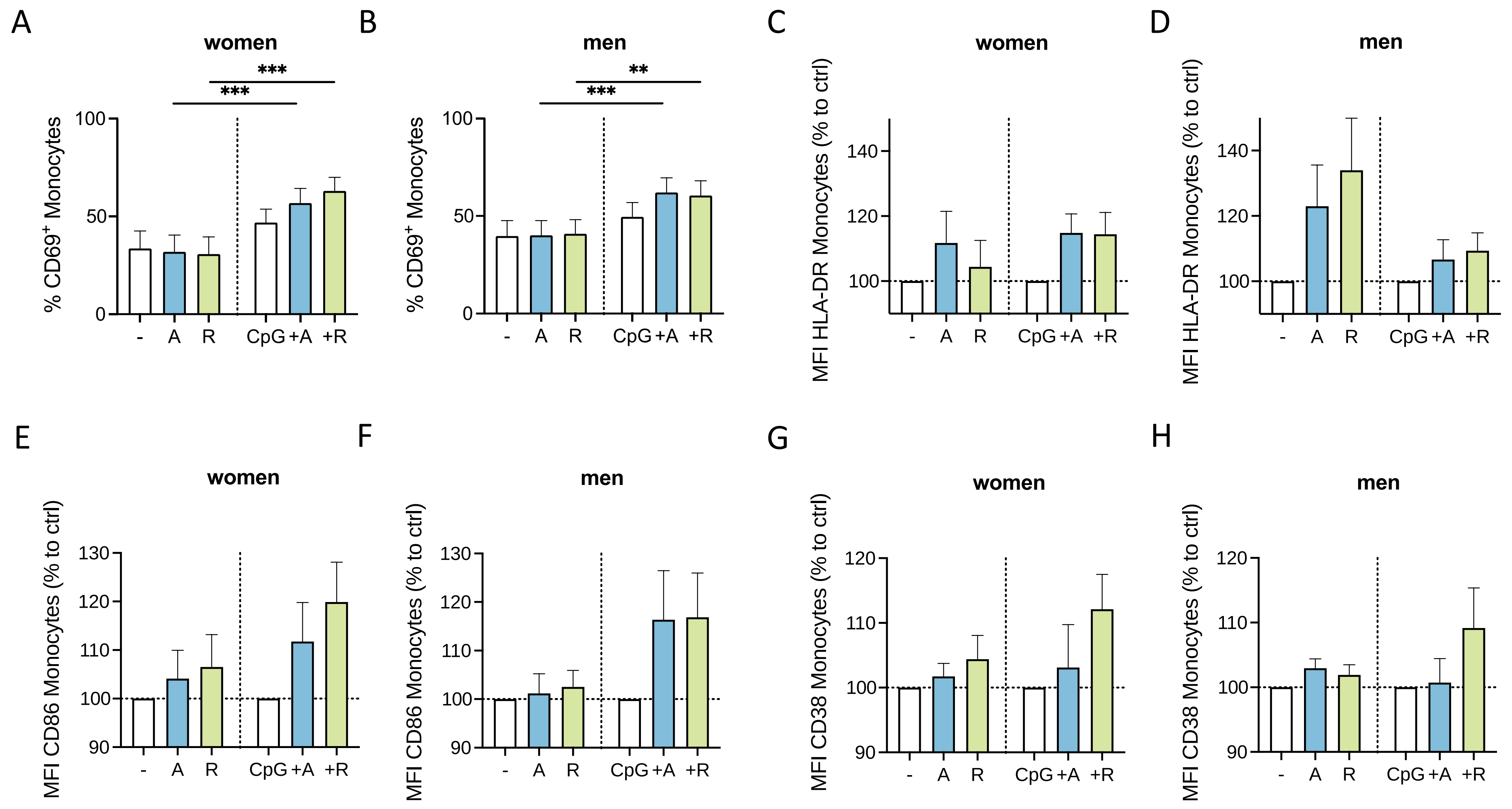

Supplementary Figure 7: Comparison of effects on activation marker expression of monocytes after DR stimulation with and without an inflammatory stimulus. A-H) Percentage of CD69+ monocytes (A, B) and expression of HLA-DR (C, D), CD86 (E, F) and CD38 (G, H) on monocytes from women (A, C, E, G) and men (B, D, F, H) after stimulation of PBMCs with A68930 (A, 10^{-7} M), Ropinirole (R, 10^{-6} M), CpG (0.195 μM) + A68930 (A, 10^{-7} M) and CpG (0.195 μM) + Ropinirole (R, 10^{-6} M) for 24 h measured via flow cytometry; normalized to unstimulated or CpG control, respectively; n=14 per condition. Wilcoxon test was used for comparison of paired data including unstimulated vs. stimulated samples; **p ≤ 0.01, ***p ≤ 0.001.

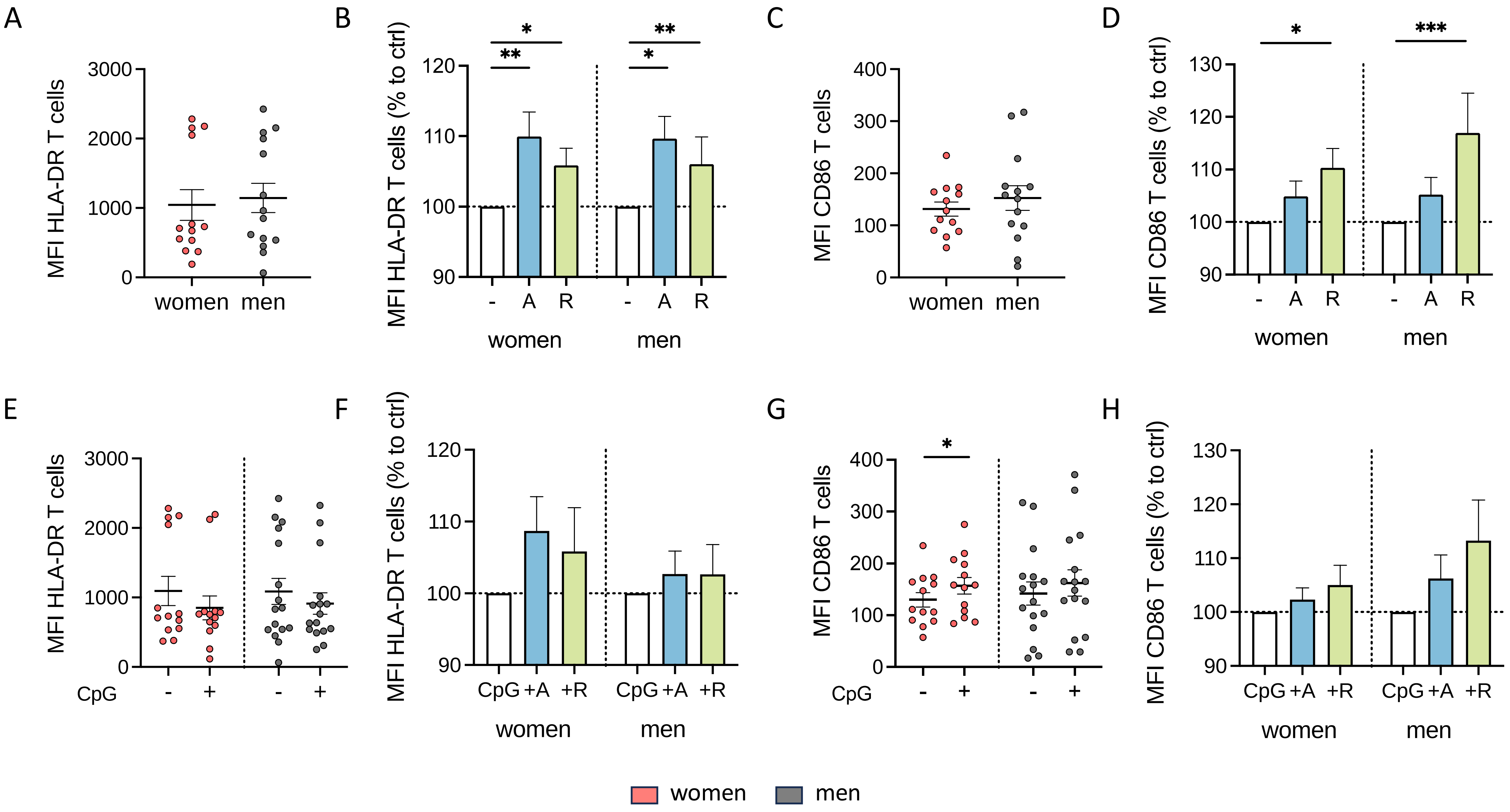

Supplementary Figure 8: Increase in HLA-DR on T cells after DR stimulation is independent of sex and inflammation. A-D) HLA-DR (A, B) and CD86 (C, D) expression on T cells from women and men after 24 h in mixed PBMC culture with or without stimulation by A68930 (A, 10^{-7} M) or Ropinirole (R, 10^{-6} M) measured via flow cytometry; B and D were normalized to unstimulated control; n=13-14 per group. **E-H**) HLA-DR (E, F) and CD86 (G, H) expression on T cells from women and men after 24 h in mixed PBMC culture with or without CpG (0.195 μM) stimulation, and after stimulation with CpG (0.195 μM) + A68930 (A, 10^{-7} M) or CpG + Ropinirole (R, 10^{-6} M) measured via flow cytometry; F and H were normalized to CpG control; n=11-12 per group. Mann-Whitney test was used for testing statistical significance between unpaired data of women and men. Wilcoxon test was used for comparison of paired data including unstimulated vs. stimulated samples; *p ≤ 0.05, **p ≤ 0.01, ***p ≤ 0.001.

Name	Company	Cat Nr
Brilliant Violet 510™ anti-human IgD	Biolegend	48220
Spark Blue™ 550 anti-human CD3	Biolegend	344851
PerCP anti-human CD4	Biolegend	317432
Brilliant Violet 711™ anti-human CD8	Biolegend	344734
PE/Fire™ 700 anti-human CD56	Biolegend	392428
PE/Cyanine7 anti-human CD27	Biolegend	356412
Brilliant Violet 650™ anti-human CD14	Biolegend	301836
APC/Cyanine7 anti-human CD19	Biolegend	302218
APC/Fire™ 810 anti-human CD38	Biolegend	356643
Dopamine Receptor D1 Antibody, PE conjugated	Bioss	10610R-PE
D2 Receptor Antibody Alexa Fluor 488®	Santa Cruz	sc-5303 AF488
DRD3 Polyclonal Antibody, Cy5 conjugated	Bioss	bs-1743R-Cy5
D4DR Antibody Alexa Fluor® 594	Santa Cruz	sc-136169 AF594
Human Dopamine D5R/DRD5 Alexa Fluor® 405-conjugated	R&D	FAB82861P
Spark NIR™ 685 anti-human CD14	Biolegend	399209
Brilliant Violet 421™ anti-human CD71	Biolegend	334122
Brilliant Violet 605™ anti-human HLA-DR	Biolegend	307640
Brilliant Violet 711™ anti-human CD69	Biolegend	310943
PE/Dazzle™ 594 anti-human CD21	Biolegend	354922
GPR30 antibody (FITC)	Biorbyt	orb15689
Androgen R/NR3C4 Alexa Fluor® 405-conjugated Antibody	R&D	FAB5876V
Estrogen Receptor alpha Antibody Alexa Fluor® 546	Santa Cruz	sc-53494 AF647
Progesterone Receptor Antibody Alexa Fluor® 594	Santa Cruz	sc-166169 AF594

Supplementary table 1: Antibodies used for flow cytometry staining of dopamine receptors, sex hormone receptors and activation markers including Annexin V.

Name	Company	Cat Nr
Brilliant Violet 510™ anti-human IgD	Biolegend	48220
Spark Blue™ 550 anti-human CD3	Biolegend	344851
PE/Fire™ 700 anti-human CD56	Biolegend	392428
PE/Cyanine7 anti-human CD27	Biolegend	356412
Brilliant Violet 650™ anti-human CD14	Biolegend	301836
APC/Cyanine7 anti-human CD19	Biolegend	302218
APC/Fire™ 810 anti-human CD38	Biolegend	356643
Brilliant Violet 711™ anti-human CD24	Biolegend	311135
FITC anti-human IL-1β	Biolegend	511705
PE anti-human IL-8	Biolegend	511408
Pacific Blue™ anti-human IL-6	Biolegend	501113
APC anti-human MCP-1	Biolegend	502611
Brilliant Violet 421™ anti-human TNF	Biolegend	502931
PE/Dazzle™ 594 anti-human IL-10	Biolegend	506811

Supplementary table 2: Antibodies used for Intracellular cytokine measurement via flow cytometry.
